# Wnt signaling pathways in biology and disease: mechanisms and therapeutic advances

**DOI:** 10.1038/s41392-025-02142-w

**Published:** 2025-04-04

**Authors:** Chen Xue, Qingfei Chu, Qingmiao Shi, Yifan Zeng, Juan Lu, Lanjuan Li

**Affiliations:** State Key Laboratory for Diagnosis and Treatment of Infectious Diseases, National Clinical Research Center for Infectious Diseases, National Medical Center for Infectious Diseases, Collaborative Innovation Center for Diagnosis and Treatment of Infectious Diseases, The First Affiliated Hospital, Zhejiang University School of Medicine, Hangzhou, China

**Keywords:** Cancer therapy, Prognostic markers

## Abstract

The Wnt signaling pathway is critically involved in orchestrating cellular functions such as proliferation, migration, survival, and cell fate determination during development. Given its pivotal role in cellular communication, aberrant Wnt signaling has been extensively linked to the pathogenesis of various diseases. This review offers an in-depth analysis of the Wnt pathway, detailing its signal transduction mechanisms and principal components. Furthermore, the complex network of interactions between Wnt cascades and other key signaling pathways, such as Notch, Hedgehog, TGF-β, FGF, and NF-κB, is explored. Genetic mutations affecting the Wnt pathway play a pivotal role in disease progression, with particular emphasis on Wnt signaling’s involvement in cancer stem cell biology and the tumor microenvironment. Additionally, this review underscores the diverse mechanisms through which Wnt signaling contributes to diseases such as cardiovascular conditions, neurodegenerative disorders, metabolic syndromes, autoimmune diseases, and cancer. Finally, a comprehensive overview of the therapeutic progress targeting Wnt signaling was given, and the latest progress in disease treatment targeting key components of the Wnt signaling pathway was summarized in detail, including Wnt ligands/receptors, β-catenin destruction complexes, and β-catenin/TCF transcription complexes. The development of small molecule inhibitors, monoclonal antibodies, and combination therapy strategies was emphasized, while the current potential therapeutic challenges were summarized. This aims to enhance the current understanding of this key pathway.

## Introduction

The Wnt signaling pathway, a highly conserved and critical regulator of diverse cellular processes, governs embryonic development, cell proliferation, differentiation, migration, and tissue homeostasis.^1–4^ Originating from the integration of the mouse breast cancer (BC) integrase-1 and Drosophila’s wingless gene, the Wnt gene unifies these related genes and proteins under the umbrella of Wnt genes.^2,5,6^ The pathway is categorized into the canonical and non-canonical branches based on β-catenin’s involvement in transcriptional activation.^7^ The canonical Wnt pathway is characterized by β-catenin’s nuclear translocation and subsequent activation of target genes through T cell factor/lymphoid enhancer factor (TCF/LEF) transcription factors, primarily driving cell proliferation. Conversely, the non-canonical Wnt pathway functions independently of the β-catenin-TCF/LEF axis, modulating cell polarity and migration, and establishing a complex, interdependent network between the two pathways.^8,9^

The Wnt signaling pathway plays a critical role in a wide range of biological processes and holds substantial significance in cellular regulation. However, its aberrant activation is also a key contributor to various cancers.^1,10–15^ Approximately 30 years ago, Vogelstein et al. first established a connection between abnormal Wnt pathway activation and the pathogenesis of colorectal cancer (CRC).^16^ Since then, extensive research has demonstrated that dysregulation of the Wnt signaling pathway is a common feature in numerous cancers, highlighting its central role in tumorigenesis.^17–21^ Mutations and abnormal activation of Wnt components can drive unchecked cell proliferation and survival, fostering tumor initiation, progression, and metastasis. For instance, mutations in the adenomatous polyposis coli (APC) gene or β-catenin are frequently observed in CRC, resulting in persistent activation of the Wnt/β-catenin pathway.^22,23^ Similarly, aberrant Wnt signaling has been implicated in liver, breast, gastric, and several other cancers.^12,24–27^ Given its critical involvement in cancer, targeting various elements of the Wnt pathway—such as Frizzled (Fzd) receptors, β-catenin, and downstream effectors—offers promising avenues for the development of novel anticancer therapies. A deep understanding of the complex mechanisms underlying Wnt signaling dysregulation in cancer cells is essential for advancing therapeutic strategies and improving patient outcomes.

Moreover, the Wnt signaling pathway engages in extensive crosstalk with various other signaling pathways, collectively orchestrating a wide array of cellular functions. This intricate network of interactions ensures precise regulation under both physiological and pathological conditions. Wnt closely interacts with pathways such as Hedgehog (Hh), Notch, Hippo, transforming growth factor-β/small mother against decapentaplegic (TGF-β/Smad), nuclear factor-kappa B (NF-κB), and phosphatidylinositol 3-kinase/protein kinase B (PI3K/AKT).^28–33^ For example, Wnt and Hh pathways collaboratively regulate growth factor expression during embryonic limb development, influencing cell differentiation and tissue morphology.^34^ Research indicates that Hh signaling can potentiate Wnt pathway activity, while Wnt signaling, in turn, modulates Hh effectors—a dynamic interplay essential in tissue regeneration and cancer progression.^35^ Additionally, Wnt signaling intersects with the Hippo pathway through β-catenin and yes-associated protein 1/transcriptional co-activator with PDZ-binding motif (YAP/TAZ) interactions, forming a complex feedback regulatory network vital for tissue size control and stem cell maintenance.^36,37^ In sum, the interaction between the Wnt pathway and other key signaling networks plays a fundamental role in regulating development, regeneration, and pathological processes. These interactions not only support normal cellular and tissue functions but also provide valuable insights into disease mechanisms and potential therapeutic targets.

This review explores the molecular mechanisms underlying Wnt pathway activation, highlighting its critical components and examining upstream factors that may influence its function. Additionally, the crosstalk between Wnt and other signaling pathways is analyzed. The regulatory mechanisms and pathological roles of Wnt signaling in various disease processes, including cardiovascular conditions, neurodegenerative diseases, metabolic disorders, autoimmune diseases, and cancer, are thoroughly discussed. Emphasis is placed on summarizing Wnt-related genes frequently mutated in cancer and investigating the mechanisms through which the Wnt pathway impacts cancer stem cells (CSCs) and the tumor microenvironment. From a therapeutic standpoint, this review provides an in-depth analysis of current strategies targeting the Wnt pathway. Ultimately, it seeks to enhance understanding of the Wnt pathway and assess the potential of Wnt-targeted therapies in advancing disease treatment.

## Signal transduction of Wnt signaling

### Canonical Wnt/β-catenin pathway

**Fig. 1:**
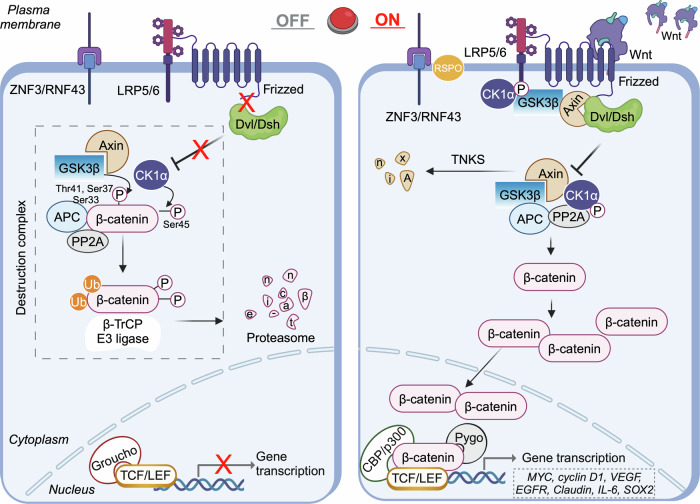
Canonical Wnt pathway signaling. Canonical Wnt pathway signaling operates in two distinct states: activation and inactivation. In the inactive state, the pathway is primarily governed by the destruction complex. Activation of the pathway necessitates the binding of Wnt ligands to their receptors. LRP lipoprotein receptor-related protein, Dvl/Dsh disheveled, GSK3β glycogen synthase kinase 3β, CK1α casein kinase 1α, APC adenomatous polyposis coli, PP2A protein phosphatase 2 A, β-TrCP β-transduction repeat-containing E3 ubiquitin-protein ligase, TCF/LEF T cell factor/lymphoid enhancer factor, TNKS Tankyrases, CBP cyclic AMP response element-binding protein, VEGF vascular endothelial growth factor, EGFR epidermal growth factor receptor, SOX sex-determining region Y-box. Image created with BioRender (https://biorender.com/)

The canonical Wnt/β-catenin signaling pathway is central to the regulation of target gene expression within the nucleus (Fig. 1).^38^ In the absence of Wnt ligands, β-catenin is phosphorylated by a multiprotein destruction complex comprising Axin, APC, glycogen synthase kinase 3β (GSK3β), casein kinase 1α (CK1α), protein phosphatase 2A (PP2A), and β-transduction repeat-containing E3 ubiquitin-protein ligase (β-TrCP).^39,40^ This phosphorylation marks β-catenin for ubiquitination, targeting it for proteasomal degradation.^41,42^ However, when Wnt proteins are present, they bind to the N-terminal cysteine-rich domain of Fzd family receptors, disrupting the formation of the Axin/GSK3β/APC complex by recruiting cytosolic disheveled (Dvl in mammals and Dsh in drosophila) proteins, thus initiating Wnt signaling.^43–45^ These Fzd receptors, characterized by their seven-transmembrane structure, belong to the G protein-coupled receptor family. To effectively propagate Wnt signaling, additional co-receptors and interactions with Wnt proteins and Fzd receptors are often necessary. Notable co-receptors include lipoprotein receptor-related protein (LRP)-5/6, receptor tyrosine kinase (RTK), and others such as RTK-like orphan receptor 2 (ROR2), GPR124, Reck, and TMEM59.^46,47^ When Wnt binds to both Fzd and LRP5/6, the phosphorylation and subsequent disruption of the destruction complex occur, facilitated by the binding of dephosphorylated Axin to the cytoplasmic tail of LRP5/6. This dephosphorylation reduces Axin’s stability and concentration, while Dvl activation through phosphorylation inhibits the GSK3β-mediated destruction complex, preventing β-catenin degradation. As a result, β-catenin accumulates in the cytoplasm, eventually translocating to the nucleus, where it associates with transcriptional coactivators (e.g., CBP/p300, BRG1, BCL9, Pygo) and TCF/LEF1, initiating the transcription of Wnt target genes.^48,49^ Besides the multiprotein destruction complex model, which stabilizes β-catenin, another model of Wnt activation involves the sequestration of GSK3 at the cell membrane into multivesicular bodies (MVBs) and lysosomes. Wnt signaling induces GSK3 sequestration from the cytoplasm into MVBs, thereby isolating the enzyme from numerous cytoplasmic substrates.^50–53^ In conclusion, the nuclear accumulation and transcriptional activity of β-catenin are critical outcomes of Wnt signaling.

### Non-canonical Wnt pathways

**Fig. 2:**
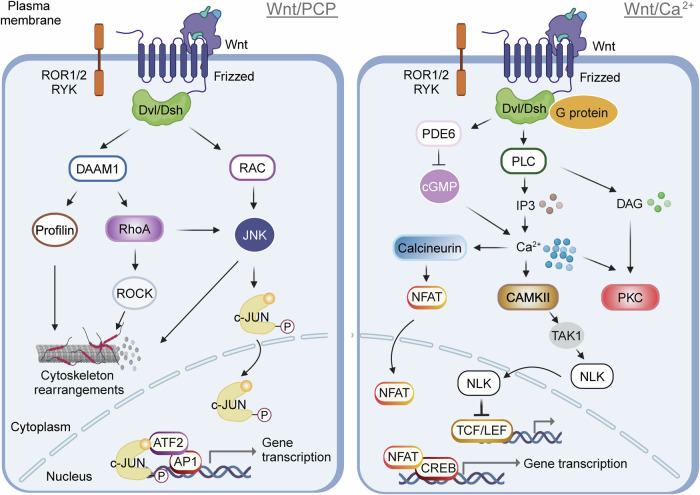
Non-canonical Wnt pathway signaling encompasses two major branches: the Wnt/PCP (Planar Cell Polarity) pathway and the Wnt/Ca^2+^ pathway. Dvl/Dsh disheveled, DAAM1 Disheveled-associated activator of morphogenesis 1, JNK Jun N-terminal kinase, ROCK Rho-associated kinase, PLC phospholipase C, IP3 inositol triphosphate, Ca2+ calcium, cGMP 3’,5’-Cyclic guanosine monophosphate, NFAT nuclear factor of activated T cells, CaMKII Calcium-calmodulin (CaM)-dependent protein kinase II, TAK1 TGF-beta-activated kinase 1, NLK Nemo-like kinase, PKC protein kinase C, TCF/LEF T cell factor/lymphoid enhancer factor, CREB cAMP-response element binding protein. Image created with BioRender (https://biorender.com/)

The non-canonical Wnt signaling pathway, also known as the non-canonical Wnt-Fzd signaling pathway, comprises two major intracellular signaling cascades: the Wnt/planar cell polarity (PCP) pathway and the Wnt/calcium (Ca^2+^) pathway (Fig. 2).^54–59^ Unlike the canonical pathway, these pathways function independently of β-catenin and are essential for regulating cell polarity, Ca^2+^ signaling, and other cellular processes. In the PCP pathway, Wnt ligands such as Wnt5a, Wnt7, and Wnt11 bind to Fzd receptors on the cell surface, initiating a signaling cascade *via* Dvl/Dsh. This activation triggers downstream signaling through Rho/Rac small GTPases and Jun N-terminal kinase (JNK).^60–64^ Specifically, Dvl/Dsh interacts with effectors like Rho-associated kinase (ROCK) *via* disheveled-associated activator of morphogenesis 1 (DAAM1), leading to the activation of RAC and subsequent JNK activation through mitogen-activated protein kinase (MAPK) pathways.^65^ The Wnt target gene naked cuticle serves as an antagonist of Wnt signaling by binding to Dsh, inhibiting β-catenin activity, and promoting JNK pathway activation. In the Wnt/Ca^2+^ pathway, Wnt proteins such as Wnt1, Wnt5a, and Wnt11 also bind to Fzd receptors, activating Dvl/Dsh. This, in turn, activates phospholipase C (PLC) through G-protein signaling, resulting in the release of intracellular Ca^2+^.^66–70^ Additionally, Dvl/Dsh can activate cGMP-specific phosphodiesterase 6, which reduces intracellular cGMP levels and further elevates cytoplasmic Ca^2+^ concentration. The increased Ca^2+^ levels enhance the phosphorylation of TCF/LEF, thereby inhibiting the canonical Wnt pathway. The Wnt/Ca^2+^ pathway is essential for early embryonic development, interneuron communication, and inflammatory responses, functioning as a G protein-dependent signaling pathway.^71^

## Key components of the Wnt signaling pathway

### Wnt ligands and receptors

The Wnt family, consisting of highly conserved and cysteine-rich proteins such as Wnt2b (Wnt13), Wnt3, Wnt3a, Wnt4, Wnt5a, Wnt5b, Wnt6, Wnt7a, Wnt7b, Wnt8a, Wnt8b, Wnt9a (Wnt14), Wnt9b (Wnt14b), Wnt10a, Wnt10b, Wnt11, and Wnt16, can be classified based on their association with specific signaling pathways.^72–75^ The Wnt1 class, including Wnt1, Wnt3, Wnt3a, Wnt8a, and Wnt8b, is linked to the canonical Wnt/β-catenin pathway, while the Wnt5a class, comprising Wnt4, Wnt5a, Wnt5b, Wnt6, Wnt7a, Wnt7b, and Wnt11, is associated with non-canonical Wnt signaling.^76–79^ In addition to Wnt proteins, various other ligands modulate Wnt signaling by interacting with Fzd and/or LRP5/6 receptors.^80^ For instance, Norrin activates the canonical Wnt pathway through Fzd4 and LRP5, while R-spondin (RSPO) enhances Wnt signaling *via* leucine-rich repeat-containing G protein-coupled receptor (LGR) 4–6.^81–83^ The secreted Frizzled-related protein (sFRP) family (sFRP1-5) inhibits Wnt signaling by either competing with Wnt for Fzd binding or forming non-functional complexes.^84,85^ The dickkopf-associated protein (DKK) family (DKK1-4) antagonizes Wnt signaling by promoting the endocytosis of LRP5/6 receptors, and sclerostin inhibits Wnt signaling, potentially through interactions with LRP5.^86–89^ It is worth mentioning that Wnt needs to be palmitoylated by the acyltransferase porcupine (PORCN) before it can be secreted and bind to the above receptors.^90^

Wnt protein transmembrane receptors primarily include Fzd and low-density LRP5/6, with additional co-receptors such as RTK and single-pass transmembrane ROR2.^91^ Fzd, a 7-transmembrane protein, is characterized by a cysteine-rich domain at its extracellular N-terminus, which is critical for binding Wnt proteins.^92^ The interaction between Wnt proteins and Fzd receptors is often mediated by RSPO1-4, LGR4-6, and ubiquitin ligases such as ZNRF3 or RNF43.^93^ In the absence of RSPO, LGR interacts with ZNRF3/RNF43, leading to the ubiquitination and subsequent degradation of Fzd receptors, effectively blocking Wnt signaling. When RSPO binds to LGR, it inhibits the activity of the ubiquitin ligases, stabilizing Fzd receptors on the cell membrane and thereby promoting Wnt signaling.^94^ The LRP gene family encodes LRP receptors, which, in conjunction with Fzd receptors, form essential Wnt receptor complexes that regulate downstream signaling pathways.^95^

### Intracellular signaling molecules

In addition to ligands and receptors, several intracellular molecules play pivotal roles in the Wnt pathway. A central component of the canonical Wnt/β-catenin pathway is the β-catenin protein, which comprises an amino terminus, a carboxyl terminus, and a central region containing 12 armadillo repeats (R1–R12).^96,97^ The amino terminus, characterized by its serine/threonine-rich residues, is subject to phosphorylation by kinases such as CK1α and GSK3β, which triggers lysine ubiquitination and subsequent proteasomal degradation. The carboxyl terminus, consisting of around 100 amino acids, is instrumental in gene transcription activation.^98^ The central armadillo repeat region is critical for β-catenin’s functionality. Recent studies have identified 20 phosphorylation sites on β-catenin. GSK3β, a serine/threonine kinase, phosphorylates the serine/threonine residues at the N-terminus of β-catenin in the absence of Wnt signaling, promoting β-TRCP-mediated ubiquitination and proteasomal degradation.^41,99–101^ CK1 initially phosphorylates β-catenin at Ser45, which is followed by GSK3β-mediated phosphorylation at Thr41, Ser37, and Ser33.^2,102–105^ Axin, a product of a murine fusion site, assembles β-catenin degradation complexes with APC, GSK3β, and CK1, and interacts with other Wnt signaling components such as Dvl and PP2A.^106–111^ The Disheveled gene, first identified in Drosophila mutants with disrupted hair and seta polarity, is essential for segment polarity during early embryogenesis.^112–114^ The Dvl/Dsh protein family, including Dsh1, Dsh2, and Dsh3, contains three conserved domains: an N-terminal DIX domain, a central PDZ domain, and a C-terminal DEP domain.^115,116^ Upon activation, Dvl/Dsh enhances GSK3β phosphorylation, inhibiting GSK3β activity, leading to the accumulation of unphosphorylated β-catenin in the cytoplasm, which subsequently translocates to the nucleus.^117,118^ In the PCP pathway, Wnt11 activates Dvl through Fzd receptors at the cell membrane, which then triggers DAAM1 activation, RhoA dissociation, and subsequent ROCK2 activation, influencing cytoskeletal dynamics.^45,119,120^ Simultaneously, Dvl activates Rac, which in turn activates JNK, modulating gene transcription.^63^ The APC protein, approximately 310 kDa in size, binds to β-catenin or axin *via* a central peptide motif, playing a pivotal role in β-catenin degradation as part of the destruction complexes (DCs).^39^ Mutations in APC, which expresses two isoforms (APC and APC2) across most organisms, lead to Wnt pathway hyperactivation in various cancers. APC2 can partially substitute for APC in mediating β-catenin degradation. Saito-Diaz et al.^121^ revealed that in APC-deficient cells, APC regulates Wnt signaling through clathrin-mediated endocytosis, independent of Wnt ligands. In addition to the components of the DCs mentioned above, there are also some functional molecules that regulate the Wnt pathway by affecting the formation of the DCs. For example, RACK1 negatively regulates the Wnt signaling pathway by stabilizing the β-catenin DCs.^122^ FAM83A directly binds to β-catenin, inhibiting the assembly of the cytoplasmic DCs, thereby inhibiting subsequent phosphorylation and degradation. FAM83A is mainly phosphorylated by B-lymphoid tyrosine kinase, a member of the SRC non-receptor kinase family, at tyrosine 138 residue in the DUF1669 domain, mediating the FAM83A-β-catenin interaction.^123^ TCF/LEF transcription factors exert bidirectional regulatory effects by binding to Groucho to suppress gene transcription or to β-catenin to enhance the transcription of downstream target genes.^98,124,125^ β-catenin/TCF-mediated transcriptional targets include matrix metalloproteinases (MMP)-7, UPAR, CD44, c-Myc, c-Jun, Fos-Related Antigen-1, Cyclin D1, PPAR-Delta, TCF1, fibronectin, gastrin, COX2, and the γ2 chain of Laminin 5.^126–130^ Table 1 provides an overview of the principal components of the Wnt signaling pathway.

**Table 1:** Main components of Wnt signaling pathway

Name	Role	Function	Refs.
Wnt [Wnt1, Wnt2, Wnt2b (Wnt13), Wnt3, Wnt3a, Wnt4, Wnt5a, Wnt5b, Wnt6, Wnt7a, Wnt7b, Wnt8a, Wnt8b, Wnt9a (Wnt14), Wnt9b (Wnt14b), Wnt10a, Wnt10b, Wnt11 and Wnt16]	Ligand	Binding with Fzd receptor complex and activates Wnt siginal pathway	^72^
PORCN	O-acyltransferase	Mediates Wnt palmitoylation	^90^
Fzds	7-transmembrane receptor	The CRD on the N-terminal side interacts with Wnt ligands, and the domain on the C-terminal side interacts with the PDZ domain of Dvl protein	^92^
LRP5/6	Receptor	Its phosphorylation is key to initiating signal transduction	^95^
Dvl/Dsh	Multifunctional phosphoprotein	It plays a key role in transmitting Wnt signals and includes three highly conserved domains: DIX, PZD and DEP	^45^
APC	Components of the “DCs”	Mediates the binding of phosphorylated β-catenin to the ubiquitin-mediated proteolytic pathway in the cytoplasm	^39^
AXIN	Components of the “DCs”	Acts as a scaffold protein and contains domains that bind to other destruction complex components	^111^
CK1α	Components of the “DCs”	In the presence of Wnt ligand, it mediates the phosphorylation of LRP6 receptor. In the absence of Wnt ligand, it induces the phosphorylation of β-catenin	^102^
GSK-3β	Components of the “DCs”	In the presence of Wnt ligand, it mediates the phosphorylation of LRP6 receptor. In the absence of Wnt ligand, it induces the phosphorylation of β-catenin	^99^
PP2A	Components of the “DCs”	Is a serine-threonine phosphatase	^40^
β-TrCP	Components of the “DCs”, F-box E3 ubiquitin ligase	Recognition of phosphorylated β-catenin	^101^
β-catenin	Core component	When the Wnt signaling pathway is activated, it enters the nucleus and interacts with the transcription factor TCF/LEF, thereby activating Wnt-regulated genes	^96^
TCF/LEF	Transcription factor	Binds to β-catenin to promote transcription of downstream target genes.	^124^
MMPs, c-Myc	Downstream target genes	Participate in various biological processes	^127^
Rho/Rac small GTPase	Small GTPase	Involved in cytoskeletal recombination	^63^
JNK	c-Jun N-terminal kinase	Influence gene expression by phosphorylating transcription factors and regulate cell polarity and movement	^65^
G protein	Signal transduction protein	After binding to the receptor, it activates PLC	^69^
Ca^2+^	Second messenger	Activates downstream effectors	^70^

*APC* adenomatous polyposis coli, *CK1α* casein kinase 1α, *CRD* cysteine-rich domain, *DCs* destruction complexes, *Dvl/Dsh* disheveled, *Fzd* frizzled, *GSK3β* glycogen synthase kinase 3β, *JNK* Jun N-terminal kinase, *LRP* lipoprotein receptor-related protein, *MMPs* matrix metalloproteinases, *PLC* phospholipase C, *PORCN* porcupine, *PP2A* protein phosphatase 2A, *TCF/LEF* T cell factor/lymphoid enhancer factor, *β-TrCP* β-transduction repeat-containing E3 ubiquitin-protein ligase

### Upstream of the Wnt signaling pathway

Dysregulation of the Wnt signaling pathway is implicated in various diseases, particularly cancer.^10,131,132^ Therefore, understanding the molecular mechanisms that control the upstream signals of the Wnt signaling cascade across different cancer types is essential for advancing effective therapeutic strategies.^133–136^ Studies have identified multiple upstream signals that regulate the Wnt/β-catenin pathway.^137,138^ In addition to proteins and other factors like NR2E3, PARP1, and USP43, several non-coding RNAs, including microRNAs (miRNAs), long non-coding RNAs (lncRNAs), and circular RNAs (circRNAs) such as miR-181a and circFBXO7, serve as key upstream regulators in disease progression.^139,140^ Moreover, paracrine Wnt activation during interactions between CSCs and M2 macrophages creates a positive feedback loop, potentially enhancing the cancer’s aggressiveness.^141^ Focal adhesion kinase, a non-receptor protein tyrosine kinase, modulates the Wnt/β-catenin pathway and plays a key role in the initiation and progression of tumors such as BC, lung cancer, and ovarian cancer (OC).^142–144^ In OC, FOXP4 directly induces the expression of protein tyrosine kinase 7, a Wnt-binding pseudokinase, leading to aberrant Wnt activation and tumor progression, offering potential therapeutic targets for resistant and recurrent cancers.^145^ Additionally, the absence of the orphan nuclear receptor NR2E3 enhances Wnt/β-catenin activation and combined with p53 inactivation, accelerates HCC progression.^146^ Studies have also shown that combining the monoclonal antibody LGR4-mAb with chemotherapeutic agents induces ferroptosis by modulating Wnt signaling, presenting promising avenues for treating hard-to-treat cancers.^147^ In lung cancer, FOXF1 deficiency decreases Wnt/β-catenin signaling in tumor vascular endothelial cells by directly activating Fzd4 transcription, significantly impacting disease progression.^148^ Mcam, a cell adhesion molecule initially identified in melanoma, also plays a role in Wnt signaling regulation.^149,150^ Recent studies have shown Mcam deficiency leads to an increase in unregulated Wnt receptor Ryk in basal cells, facilitating Wnt5a-Ryk interaction and promoting unchecked breast epithelial cell proliferation, potentially contributing to tumorigenesis.^151^ In CRC, the lncRNA β-secretase 1 antisense RNA (BACE1-AS) is linked to poor prognosis.^152,153^ Dysregulation of BACE1-AS can activate the Wnt pathway *via* Tuftelin 1, driving liver metastasis in metastatic CRC cases.^154^

The studies explore the molecular mechanisms governing upstream signaling within the Wnt pathway, shedding light on the roles of key regulatory molecules in the Wnt cascade across various diseases. These insights not only deepen our understanding of disease progression but also present new opportunities for developing targeted therapeutic interventions aimed at modulating the Wnt signaling pathway.

## Crosstalk of the wnt signaling pathway

The conserved Wnt pathway plays a key role in maintaining stem cell pluripotency and determining cell differentiation outcomes. This developmental cascade seamlessly integrates signals from other key pathways, including retinoic acid, fibroblast growth factor (FGF), Notch, Hh, TGF-β, NF-κB, and bone morphogenetic protein (BMP), across various cell types and tissues. The following discussion explores the interactions between the Wnt pathway and these signaling networks to deepen our understanding of their collective influence on disease development and progression.

### Wnt and Notch signaling pathway

Both Wnt and Notch signaling pathways are central to regulating a wide array of developmental processes, tissue homeostasis, and disease pathogenesis.^155^ Their crosstalk involves complex molecular interactions that orchestrate cell fate determination, proliferation, differentiation, and tissue patterning. The Notch pathway, another highly conserved signaling mechanism, regulates multiple cellular processes, including fate determination, differentiation, and proliferation. Notch receptors (Notch1-4 in mammals) are transmembrane proteins characterized by extracellular domains containing EGF-like repeats and LIN12-Notch repeats (LNR), which mediate ligand binding and prevent premature activation of the receptor.^156,157^ These receptors interact with ligands from the Delta-like (DLL1/3/4) and Jagged (JAG1/2) families on adjacent cells. Upon ligand binding, the Notch receptor proceeds proteolytic cleavage, releasing the Notch intracellular domain (NICD), which then translocates to the nucleus.^158,159^ In the nucleus, NICD interacts with transcriptional regulators such as CBF1/suppressor of hairless/Lag1 (CSL, also named RBPJ) to trigger the transcription of Notch target genes.^160,161^ The most well-known Notch/RBPJ target genes include basic helix-loop-helix (BHLH) transcription factors, such as Hairy and Enhancer of Split (Hes) and Hes-related factors related to the YRPW motif.^162,163^

The interplay between the Wnt and Notch pathways exerts significant influence over each other, with components from one pathway directly modulating the other.^164^ For instance, β-catenin engages with Notch signaling elements like NICD and CSL, thereby altering the transcription of Notch target genes, which in turn impacts cell fate determination and differentiation. Both pathways are involved in reciprocal regulation, as they control the expression of genes that either activate or suppress the opposing pathway. For example, the Notch target gene Hes1 encodes a powerful BHLH transcriptional repressor, whose expression is modulated by β-catenin-mediated Wnt signaling.^165^ Conversely, NICD can complex with β-catenin, inhibiting its binding to target sites and redirecting β-catenin to NICD/RBPJ target sites, thereby suppressing β-catenin activity.^164^ Moreover, the Wnt/Ca^2+^ pathway interacts with Notch signaling; Wnt5a activation of CaMKII leads to the phosphorylation of SMRT, a co-repressor of RBPJ interaction, on serine-1407, which enhances the promoter activity of Notch-responsive genes.^166^ GSK3β and Axin serve as vital mediators connecting these two signaling pathways.^167–169^ The interactions between Wnt and Notch pathways are integral to various developmental processes, including embryogenesis, tissue patterning, and organogenesis.^170^ The coordinated regulation of these pathways is essential for the correct specification of cell fate, differentiation, and morphogenesis. In adult tissues, the crosstalk between Wnt and Notch pathways plays a pivotal role in maintaining tissue homeostasis by regulating stem cell maintenance, proliferation, and differentiation. For instance, in the skin, Notch1 activation suppresses the Wnt pathway by downregulating the expression of Wnt ligand genes. Additionally, p21 acts as a negative regulator of Notch1’s activation of downstream Wnt transcription, thereby linking the Notch and Wnt pathways in keratinocyte growth control.^171^ Localized interactions between these pathways also promote the regeneration of sensory hair cells.^172^ Kwon et al. demonstrated that Notch negatively regulates active β-catenin levels in stem and progenitor cells. Inhibition of Notch signaling drives intestinal stem cells to differentiate into secretory cells while reducing differentiation into nutrient-absorbing cells.^173^ Furthermore, blocking the Notch receptor with specific antibodies disrupts the inhibition of the Wnt pathway, leading to impaired intestinal stem cell function. This underlines the critical physiological role of Wnt and Notch signaling interactions in sustaining stem cell activity and maintaining the balance of differentiation.^174^ However, dysregulation of this crosstalk can contribute to tissue dysfunction and the pathogenesis of various diseases. Abnormal activation or inhibition of the Wnt and Notch pathways is implicated in numerous human conditions, including cancers, neurodegenerative disorders, and developmental syndromes.^175^ Rodilla et al. identified Notch as a key regulator of nuclear β-catenin-induced tumorigenesis.^176^ In this context, Notch activation, mediated by β-catenin-induced upregulation of JAG1, is essential for intestinal tumorigenesis, a mechanism also observed in human tumors from patients with familial adenomatous polyposis. Additionally, enhanced Wnt signaling induces the oncogenic transformation of human mammary epithelial cells through a Notch-dependent mechanism, and Notch inhibitors can prevent this transformation, underscoring the necessity of Wnt/Notch interactions in disease progression.^177,178^ Notch2 is known to control Wnt signaling in leukemia, and Notch2 activation within the microenvironment is necessary for canonical Wnt signaling activation in tumor cells.^179^ A thorough understanding of these pathway interactions is essential for the development of targeted therapeutic strategies.

### Wnt and Hedgehog signaling pathway

The Hedgehog (Hh) signaling pathway represents a key developmental cascade that governs embryonic patterning, tissue homeostasis, and stem cell maintenance. First identified in Drosophila, the fundamental components of the Hh pathway are preserved across vertebrates. In mammals, the singular Hh gene found in invertebrates has diversified into three paralogous genes: Sonic Hedgehog (SHH), Indian Hedgehog (IHH), and Desert Hedgehog (DHH).^180^ In the absence of an Hh ligand, the transmembrane receptor Patched (PTC) suppresses the activity of the transmembrane protein Smoothened (SMO). SMO, a 7-pass transmembrane protein like the Fzd receptor in the Wnt pathway, becomes de-repressed upon Hh ligand binding to PTC, initiating downstream signaling cascades. These cascades include the dissociation of SUFU and COS proteins, leading to the activation and nuclear translocation of the glioma-associated oncogene (GLI) family transcription factors, which regulate genes responsible for cell proliferation, survival, and differentiation. The GLI family comprises multifunctional transcription factors GLI1, GLI2, and GLI3, each containing DNA-binding sites and a C-terminal activation domain, with GLI2 and GLI3 also possessing an N-terminal inhibitory domain.^181^ Mirroring the Wnt signaling pathway, the Hh pathway operates in both canonical and non-canonical forms, and the two pathways exhibit several shared features.^182^ In their inactive states, essential transcription factors within both pathways undergo ubiquitination and are targeted for proteolysis, resulting in their inactivation. Upon activation, both pathways involve the disassembly of cytosolic protein complexes, inhibition of ubiquitination, and the subsequent release of active transcription factors. Additionally, kinases such as GSK3β play a critical role in the signaling mechanisms of both pathways.^183–185^

Since 2004, researchers have recognized IHH as an antagonist of Wnt signaling in the differentiation of colon epithelial cells.^186,187^ In vivo, Hh signaling restricts the expression of Wnt targets to the basal regions of colonic crypts. Introducing IHH into colon cancer cells reduces the components of the nuclear TCF4-β-catenin complex, thereby diminishing their function in Wnt signaling. Notably, IHH expression is suppressed in polyps from individuals with familial adenomatous polyposis, revealing a novel Wnt-Hh axis in the differentiation of colon epithelial cells. Further investigations have demonstrated that components of the Wnt pathway can modulate Hh signaling and vice versa. For instance, Wnt inhibits SHH-driven tumorigenesis, while both GSK3β and CK1α phosphorylate GLI3, leading to its ubiquitination by β-TrCP and subsequent inhibition.^188^ These kinases serve as negative regulators of both the β-catenin and GLI protein families.^189,190^ Inhibition of SMO has been shown to decrease active β-catenin levels.^191^ The crosstalk between Wnt and Hh signaling is predominantly mediated by sFRP-1, a key negative regulator of both the Wnt/β-catenin and Hh/GLI pathways through its inhibition of fusion kinases.^192^ Hh signaling induces sFRP-1 expression, thereby diminishing β-catenin transcriptional activity. Downstream of Hh, Wnt signaling is essential for osteoblast maturation during endochondral bone formation following Osterix expression.^193^ The inhibition of Hh signaling by cyclopamine further supports this, as it leads to reduced transcriptional activity in colon cancer cell lines.^194^ The interplay between Hh and Wnt pathways significantly impacts cancer recurrence, invasion, and metastasis, suggesting that disrupting this interaction could impede cancer progression.^195–197^ For example, Wnt/β-catenin signaling induces RNA-binding proteins (RBPs) that stabilize GLI1 mRNA, thereby enhancing Hh signaling and promoting CRC cell survival.^198^ N-myc, a pivotal Wnt target, is regulated by SHH and is essential for N-myc expression and stability, establishing its connection to medulloblastoma. GLI1 induces sFRP-1 expression in gastric cancer cell lines, where Hh signaling upregulates sFRP-1 to inhibit Wnt signaling.^199,200^ Ma et al. explored the roles of SHH and Wnt in tumor regeneration following radiotherapy and discovered that Wnt signaling was suppressed, as evidenced by low levels of activated nuclear β-catenin, while Hh, GLI1, and sFRP-1 levels were elevated.^201^ This indicates that SHH activation downregulates Wnt signaling, facilitating tumor cell line regeneration.

### Wnt and the TGF-β signaling pathway

**Fig. 3:**
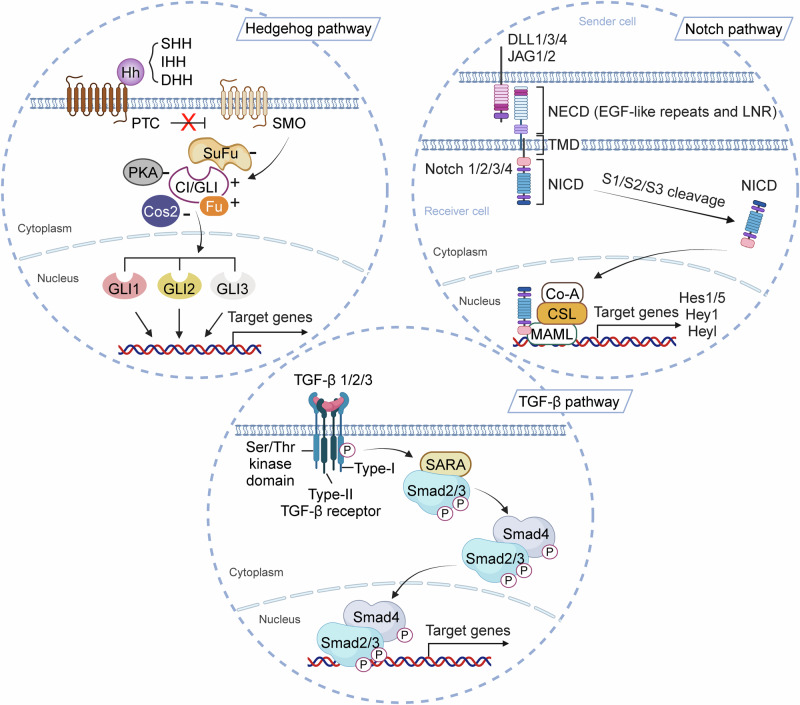
Overview diagram of Hedgehog pathway, Notch pathway and TGF-β pathway. Hh Hedgehog, SHH Sonic Hedgehog, IHH Indian Hedgehog, DHH Desert Hedgehog, PTC Patched, SMO Smoothened, Cos2 Costal 2, Fu fused, Ci/Gli Cubitus interruptus/Gli, JAG Jagged, DLL Delta-like, NICD Notch intracellular domain, NECD Notch extracellular domain, EGF epidermal growth factor, LNR LIN12-Notch repeat, TGF-β transforming growth factor-β, SARA Smad anchor for receptor activation. Image created with BioRender (https://biorender.com/)

The TGF-β pathway orchestrates numerous vital processes.^202^ In humans, the TGF-β family consists of 33 proteins, including 3 TGF-β isoforms (TGF-β1/2/3), 10 BMPs, 11 growth and differentiation factors, as well as additional members like activin, nodal, inhibin, and AMH/MIS. These proteins are dimeric-secreted polypeptides that, upon ligand binding, initiate signaling cascades.^203,204^ Specifically, type II TGF-β receptors recruit and phosphorylate type I TGF-β receptors, which in turn phosphorylate downstream Smad proteins. These phosphorylated Smads then form complexes with co-Smad proteins, which translocate to the nucleus to regulate the expression of target genes involved in various cellular functions.^205–207^ Smad proteins, whose name derives from Sma in C. elegans and Mad in Drosophila, serve as key signal transducers downstream of TGF-β family receptors.^208–210^ In mammals, there are eight Smad proteins (Smad1 to Smad8), categorized into receptor-regulated Smads, common-mediator Smads, and inhibitory Smads, reflecting their distinct roles within the TGF-β signaling pathway (Fig. 3).

Both the Wnt and TGF-β pathways converge on key target genes that govern cell fate determination, proliferation, and differentiation, suggesting potential mechanisms of crosstalk. Labbé et al. identified four such genes in normal mouse epithelial cells—CTGF, ROBO1, GPC1, and INHBA—shared by both pathways.^211^ Transgenic mouse models revealed that many of these genes are overexpressed in breast and colon tumors, and inactivation of the TGF-β pathway resulted in reduced expression of some genes and delayed tumor formation, underscoring the synergistic role of TGF-β and Wnt in promoting tumorigenesis. In zebrafish, these pathways jointly regulate posterior mesoderm formation by cooperatively activating genes such as TBX6.^212^ In the dorsal telencephalon, Wnt and BMP signals directly target EMX2, regulating its hierarchical expression in a coordinated manner.^213^ Crosstalk between the Wnt and TGF-β pathways modulates cellular responses to external stimuli, fine-tuning growth, differentiation, and migration decisions during development and tissue homeostasis.^214^ Feedback mechanisms are essential in this interaction; activation of one pathway may induce negative regulators of the other, resulting in feedback inhibition and ensuring the precise control of signaling activities to achieve specific cellular outcomes. For instance, in mouse embryos, Wnt signaling directly regulates BMP target gene MSX2 or induces BMP ligands, thereby influencing ectoderm and neural crest cell fate.^215^ Additionally, human bronchial epithelial cell-derived extracellular vesicles, through miRNA-mediated inhibition of TGF-β-Wnt crosstalk, exhibit potential as an anti-fibrotic treatment for idiopathic pulmonary fibrosis.^216^ Wnt signaling also promotes tooth germ development *via* YAP1-TGF-β signaling.^217^ Compound heterozygous mice lacking Smad4 and APC demonstrate a heightened susceptibility to intestinal or pancreatic tumors compared to mice lacking APC alone, while Smad2 deletion accelerates colon cancer progression in APC-deficient mice.^218–220^ The interplay between TGF-β/Smad3 and Wnt/β-catenin pathways promotes vascular smooth muscle cells (VSMCs) proliferation.^221^ Mechanistically, the TGF-β/BMP and Wnt pathways coordinate development and homeostasis by regulating stem cell self-renewal and differentiation. In human embryonic stem cells, BMP, together with FGF2, induces mesoderm differentiation, dependent on TGF-β or Wnt signaling.^222^ In transformed mammary epithelial cells, TGF-β and Wnt signaling synergistically activate the EMT program and maintain the stem cell state in an autocrine manner.^223^ Additionally, in prostate cancer, Fzd8 integrates Wnt-11 and TGF-β signaling, colocalizing and coimmunoprecipitating with Wnt-11 to enhance ATF2-dependent transcription. Silencing Fzd8 reduces prostate cancer cell migration, invasion, and TGF-β/Smad signaling, indicating that targeting Fzd8 could inhibit aberrant Wnt and TGF-β signaling in prostate cancer.^224^ Furthermore, PP2 mitigates osteoarthritis progression by inhibiting Wnt/β-catenin and activating the TGF-β/Smad pathway.^225^ In bone metastasis, TGF-β-induced DACT1 biomolecular condensates repress Wnt signaling.^226^

### Wnt and FGF signaling pathway

**Fig. 4:**
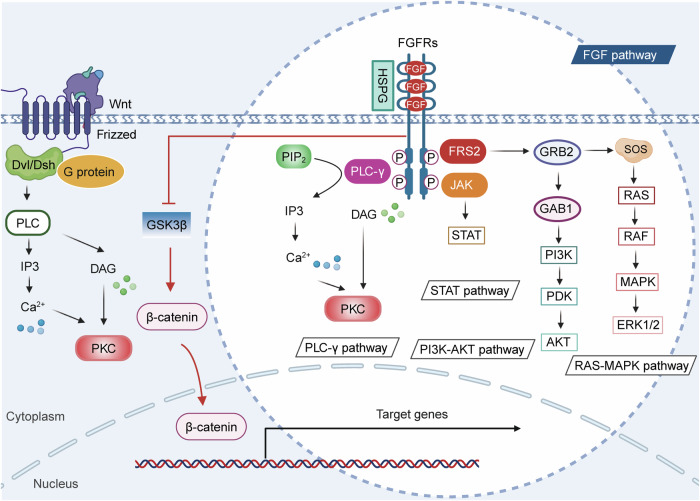
Crosstalk between Wnt and FGF signaling pathways. Dvl/Dsh disheveled, PLC phospholipase C, IP3 inositol triphosphate, Ca2+ calcium, DAG diacylglycerol, PKC protein kinase C, GSK3β glycogen synthase kinase 3β, PIP2 phosphatidylinositol bisphosphate, FRS2 fibroblast growth factor receptor substrate 2, JAK Janus kinase, STAT signal transducer and activator of transcription, GRB2 growth factor receptor-bound protein 2, GAB1 GRB2-associated binder 1, PI3K phosphatidylinositol 3-kinase, PDK phosphoinositide-dependent kinases, AKT protein kinase B, SOS son of sevenless, MAPK mitogen-activated protein kinases, ERK extracellular-signal-regulated kinases. Image created with BioRender (https://biorender.com/)

The FGF family comprises 22 distinct secreted ligands, encompassing FGF1-14 and FGF16–23, which interact with four highly homologous tyrosine kinase receptors: FGFR1, FGFR2, FGFR3, and FGFR4. Upon binding, these interactions prompt FGFR dimerization and subsequent phosphorylation of tyrosine residues, initiating a cascade of intracellular signaling pathways.^227,228^ The specificity and functional diversity of FGF ligands are partly governed by their unique amino acid sequences and structural characteristics. FGF binding to FGFR typically necessitates the presence of heparan sulfate proteoglycans as cofactors, which enhance ligand-receptor affinity. The principal downstream signaling pathways activated by FGF include the RAS-MAPK, PI3K-AKT, PLC-γ, and signal transducer and activator of transcription (STAT) pathways.^229–232^ FGF signaling triggers the RAS-MAPK cascade by activating the tyrosine kinase activity of FGFR, culminating in the activation of ERK1/2, which plays a pivotal role in regulating cell proliferation, differentiation, and survival. Concurrently, FGF activates PI3K, leading to the activation of AKT, a key regulator of cell survival, metabolism, and growth. Additionally, phosphorylation of tyrosine residues in FGFR’s C-terminal region recruits and activates PLC-γ, facilitating the conversion of phosphatidylinositol bisphosphate (PIP_2_) into diacylglycerol (DAG) and inositol triphosphate (IP3). This activation of PLC-γ increases intracellular Ca^2+^ levels and triggers protein kinase C (PKC), which is essential for cell migration and differentiation. Furthermore, FGF signaling can activate STAT transcription factors *via* the JAK pathway, contributing to the regulation of immune responses and cell survival (Fig. 4).

Research indicates that the FGF signaling pathway not only interacts with the canonical Wnt signaling cascade to regulate the transcription of target genes but also engages in crosstalk with non-canonical Wnt signals, influencing cell phenotype and migration. FGF signaling enhances β-catenin stability by inhibiting GSK3β, thereby amplifying Wnt pathway activity. Additionally, FGF modulates the expression of Wnt pathway inhibitors, such as Dkk1, indirectly influencing Wnt signal strength. Through FGFR signaling, FGF induces tyrosine phosphorylation of C-terminal β-catenin, releasing it from adherens junctions, while concurrently reducing serine/threonine phosphorylation at the N-terminal, thereby preventing β-catenin from proteasomal degradation. This positive feedback mechanism ultimately strengthens the canonical Wnt signaling cascade.^233–235^ In liver fibrosis, FGF9 potentially accelerates the disease by upregulating hepatocyte β-catenin signaling, which may increase intrahepatic extracellular matrix (ECM) production.^236^ FGF2 signaling *via* the MAPK pathway also modulates the extent of LRP6 phosphorylation at its intracellular PPPS/TP motif, thereby further enhancing the canonical Wnt cascade.^237^ FGF-induced cell proliferation contributes to this process by promoting LRP6 phosphorylation *via* the cyclin Y/PFTK complex during the G2/M phase of the cell cycle. Moreover, FGF signaling through the RAS-MAPK pathway regulates TCF transcription *via* Ets, which is essential for the induction and differentiation of pigment cells from early gastrulation to neural tube closure during S. luteus embryogenesis. FGF18 and FGF20, transcriptionally activated by the β-catenin-TCF/LEF complex, are directly upregulated by the canonical Wnt signaling cascade.^238,239^ FGF7 and FGF10, signaling through FGFR2b to SRC, induce tyrosine phosphorylation of F-actin binding proteins involved in endocytosis, clathrin-dependent internalization, and the polarization of FGFR2b to the leading edge, thus regulating cell migration.^240^ Furthermore, FGF signaling *via* the PI3K-AKT pathway downregulates GSK3β activity, leading to EMT through increased stability and nuclear translocation of SNAI1.^241^ Additionally, the Hh-TGF-β signaling axis induces the transcriptional upregulation of Wnt5a, a non-canonical Wnt ligand. Wnt5a, in turn, upregulates SNAI1 protein *via* PKC and promotes cancer cell migration and invasion by activating non-canonical Wnt signaling pathways.

### Wnt and NF-κB signaling pathway

The NF-κB family of transcription factors plays a pivotal role in a wide array of biological processes, including immune responses, inflammation, cell growth, survival, and development.^242–244^ In mammals, this family consists of five members: NF-κB1 (p105/p50), NF-κB2 (p100/p52), ReLA (p65), ReLB, and c-REL.^245–247^ NF-κB signaling operates through two primary pathways: canonical and non-canonical.^248^ Canonical NF-κB signaling is activated by stimuli such as tumor necrosis factor-alpha (TNF-α), interleukin-1 beta (IL-1β), lipopolysaccharide (LPS), and antigens, which engage cell surface receptors and initiate NF-κB activation *via* a series of intermediary proteins.^249–251^ In contrast, the non-canonical NF-κB signaling pathway, activated by factors such as B lymphocyte activating factor, CD40 ligand, and lymphotoxin β.^242,252–254^ NF-κB signaling is not isolated in its regulation of physiological and pathological processes; rather, it interacts with other molecules and pathways, both directly and indirectly, contributing to a complex network of signaling interactions that fine-tune its regulatory effects across diverse biological functions.

Various signaling pathways converge to form a complex signal transduction network characterized by intricate regulatory mechanisms.^255^ Among them, the Wnt signaling pathway has significant potential for interacting with the NF-κB pathway, thereby influencing diverse biological processes such as cell proliferation, differentiation, survival, apoptosis, and tumorigenesis.^256^ Canonical Wnt ligands, like zebrafish Wnt8a and Xenopus Wnt11, initiate β-catenin signaling in the dorsal embryonic region, promoting dorsal organizer formation.^257^ In Drosophila embryos, NF-κB activation through Toll-like receptors (TLR) is essential for dorsal-ventral patterning, a process modulated by Wnt-mediated negative feedback that fine-tunes TLR/NF-κB signaling.^258,259^ Elevated levels of Wnt2 and Wnt4 activate the β-catenin/NF-κB signaling pathway, contributing to cardiac fibrosis *via* the collaboration of Fzd4/2 and LRP6 in fibroblasts, which may exacerbate outcomes in patients with acute myocardial infarction.^260^ The Wnt inhibitor LGK974 reduces proinflammatory cytokine production by modulating Wnt/β-catenin and NF-κB interaction, decreasing cytokine storms, liver damage, and mortality in LPS-induced endotoxemia in mice.^261^ The β-catenin/TCF signaling pathway is critical in both normal embryonic development and cancerous transformations in various human cells.^262,263^ It enhances the expression of β-transducing repeat-containing protein, which accelerates β-catenin degradation and subsequently activates NF-κB-dependent transcription.^264^ A recent study found that the Wnt/β-catenin pathway aids recovery from bladder injury in interstitial cystitis/bladder pain syndrome (IC/BPS) by downregulating NF-κB, reducing oxidative stress-induced ferroptosis, and improving IC/BPS symptoms.^265^

In contrast to rodent models, the Wnt/β-catenin pathway serves as a key mitogenic driver in adult primary human hepatocytes. Recent research highlights that inhibition of TGFβ and activation of NF-κB-mediated inflammatory signaling are critical for shifting human cells from quiescence to regeneration, thus enabling Wnt/β-catenin-induced cell proliferation.^266^ CSCs are instrumental in tumor initiation and progression.^267^ Activation of TLR3, both in vitro and in vivo, promotes the transition of BC cells toward a CSC phenotype.^267,268^ Notably, TLR3 does not rely solely on the conventional NF-κB pathway to enrich CSCs; both the β-catenin and NF-κB pathways are activated in tandem. Activation of either pathway alone proved insufficient to induce the CSC phenotype, underscoring the need for simultaneous Wnt/β-catenin and NF-κB pathway engagement in CSC enrichment.^269^

Beyond these important signaling pathways, Wnt signaling intersects with numerous others. For instance, PI3K/Akt signaling interacts with Wnt signaling in regulating glucose metabolism, protein synthesis, and cell cycle progression, particularly in tumor cells.^270–272^ The Hippo signaling pathway, when active, inhibits YAP/TAZ activity through phosphorylation, reducing its association with β-catenin and thereby suppressing Wnt signaling. Conversely, Wnt signaling can influence Hippo pathway activity by regulating upstream proteins like Merlin and Lats.^273–275^ The MAPK/ERK pathway impacts Wnt signaling by modulating critical proteins such as Axin and Dvl, and further amplifies Wnt signaling’s downstream effects by regulating transcription factors like c-Myc and Cyclin D1.^276–278^ In conclusion, the Wnt signaling pathway intricately regulates cell proliferation, differentiation, migration, and metabolism through complex interactions with multiple signaling pathways. These interactions are pivotal in organismal development, tissue homeostasis, and disease progression. A comprehensive understanding of these mechanisms is essential for deciphering cell behavior and devising innovative therapeutic strategies.

## Biology of the wnt signaling pathway

### Mutation of Wnt pathway genes

In 1982, R. Nusse and H.E. Varmus successfully cloned the first Wnt gene in mouse BC cells, marking the beginning of a profound exploration into the Wnt signaling pathway.^279^ Over the years, researchers have identified key components of this pathway, leading to a comprehensive understanding of its mechanisms. Recent studies have revealed that mutations in Wnt pathway genes are prevalent across various cancer types, which is unsurprising given Wnt signaling’s pivotal role in epithelial stem cell activity. Mutation-induced activation of Wnt signaling is a frequent oncogenic event, driving sustained cell self-renewal and proliferation, and potentially contributing to drug resistance. Among the Wnt pathway components, APC stands out as the most frequently mutated gene. In 1991, multiple research groups first identified APC as a critical gatekeeper gene, demonstrating that its inactivation leads to familial adenomatous polyposis, a syndrome characterized by widespread intestinal polyps and a high risk of CRC.^280–282^ APC mutations are also common in sporadic tumors, often occurring in the early stages of tumorigenesis.^283^ The primary mechanism through which the Wnt pathway is activated involves the loss of APC’s negative regulatory function. APC, by forming complexes with Axin and GSK3β, promotes the phosphorylation, ubiquitination, and subsequent degradation of β-catenin, thereby preventing its nuclear accumulation and inhibiting aberrant Wnt signaling. Beyond its role in Wnt regulation, APC interacts with the cytoskeleton, playing a vital role in cell adhesion, migration, and polarity, which are essential for maintaining tissue structure and function.^284^ Mutations in the APC gene, often missense, nonsense, insertions, or deletions, typically result in loss of function or truncation of the APC protein, leading to abnormal Wnt activation and tumorigenesis. Another key negative regulator in the Wnt pathway is RNF43, an E3 ubiquitin ligase that inhibits Wnt signaling by ubiquitinating Fzd receptors, thus impeding Wnt signal transduction.^285^ Inactivating mutations in RNF43 were first identified in pancreatic cancer, and they are present in approximately 10–20% of pancreatic ductal adenocarcinomas (PDAC).^286^ Additionally, RNF43 mutations occur in over 18% of colorectal and endometrial cancers.^286,287^ CRC with RNF43 mutations are highly dependent on Wnt secretion, rendering them particularly susceptible to therapies targeting Wnt secretion.^288^ Moreover, mutations in RNF43 and KRAS exhibit synergistic effects in CRC progression, with Wnt signaling activated by RNF43 mutations enhancing tumor growth and recurrence rates.^289^ RNF43 mutations are also reported in gastric cancer, adenomas from patients with Lynch syndrome, and ovarian clear cell carcinoma.^290–292^ These mutations are especially enriched in gastric cancers with microsatellite instability, contributing to resistance against DNA damage response therapies such as radiotherapy and chemotherapy. ZNRF3, another E3 ubiquitin ligase, was initially reported to have inactivating mutations in adrenocortical cancer.^293^ In human cancers, RNF43 mutations are primarily truncating and missense, whereas ZNRF3 mutations tend to be missense mutations and deletions. RSPO translocations and fusions, which occur in 4–18% of gastric, ovarian, and endometrial cancers and in about 9% of CRC cases, are mutually exclusive with APC mutations.^294^ Unlike APC mutations, which are early events in tumorigenesis, RNF43 mutations are considered late-stage events that drive the progression of adenomas to carcinomas, often associated with lower levels of Wnt pathway activation.^131^ In the context of adult stem cell niches, the negative feedback from RNF43 and ZNRF3 is partially counterbalanced by RSPO family proteins. RSPOs promote the removal of RNF43 and ZNRF3 from the cell surface by forming complexes with LGR and RNF43 or ZNRF3. Additionally, some RSPO family members facilitate the clearance of ZNRF3 and RNF43 *via* an LGR-independent mechanism involving heparan sulfate proteoglycans. This RSPO-mediated activity results in the accumulation of Wnt receptors on the cell surface, leading to elevated β-catenin-mediated transcription necessary for stem cell maintenance.

AXIN is another key component frequently mutated in the Wnt signaling pathway. As a scaffold protein within the destruction complex, AXIN enhances the complex’s cytoplasmic activity, thereby downregulating β-catenin-mediated transcription. Mutations in AXIN1 and AXIN2 significantly disrupt Wnt signaling, impairing its regulatory function. AXIN1 mutations were first identified in hepatocellular carcinoma (HCC), where loss-of-function mutations are present in approximately 11% of cases.^295^ Studies have shown that adenovirus-mediated transfer of wild-type AXIN1 can induce apoptosis in liver and CRC cells, a process hindered by mutations in APC, CTNNB1, or AXIN1. In HCC, these mutations are associated with reduced infiltration of CD4^+^ and CD8^+^ T cells, contributing to immune evasion.^296,297^ Furthermore, a novel AXIN1 mutation has been identified in advanced prostate cancer.^298^ Pan et al. reported AXIN1/2 mutations in 4 out of 70 patients with gastric cancer, all of whom exhibited nuclear β-catenin expression.^299^ Inherited AXIN2 mutations are known to increase susceptibility to colon cancer, particularly in tumors with high-frequency microsatellite instability.^300^ AXIN2 mutations have also been documented in breast and liver cancers, among others, with germline mutations linked to increased risks of lung cancer, BC, and CRC. However, the precise role of AXIN2 variants in tumorigenesis remains to be fully elucidated. GSK3β, another principal component of the destruction complex, has been shown to drive hematopoietic stem cells into a precancerous state when deleted, and its deletion promotes acute myeloid leukemia (AML) progression when GSK3A is absent.^301^ Activating mutations in β-catenin (encoded by CTNNB1) are also central to cancer progression. CTNNB1, located on chromosome 17q21.32, encodes β-catenin, a pivotal regulator of the Wnt pathway. Mutations in CTNNB1 lead to the abnormal stabilization and accumulation of β-catenin, triggering the activation of Wnt signaling and the development of various cancers. In HCC, activating mutations in CTNNB1 are observed in 28–40% of cases. Research by Zhang et al. demonstrated that mutant activated β-catenin not only initiates liver tumorigenesis but also exacerbates liver cancer progression, particularly when combined with TP53 deletion or hepatitis B virus infection.^302^ Targeting β-catenin-activated AKT2-CAD-mediated pyrimidine synthesis has been suggested as a potential therapeutic approach for liver cancer. Cai and colleagues proposed that targeting MMP-9 in CTNNB1 mutant liver cancers could restore CD8^+^ T cell-mediated antitumor immunity and enhance the efficacy of anti-PD-1 therapy. CTNNB1 mutations have been identified as potential prognostic markers and therapeutic targets in HCC.^303^ In medulloblastoma, CTNNB1 mutations are common (12%), particularly in pediatric cases, where they are associated with nuclear β-catenin positivity and a favorable prognosis.^304,305^ CTNNB1 exon 3 mutations are implicated in driving low-grade endometrioid cancer, and nearly half of CRC with wild-type APC harbor CTNNB1 mutations, with nuclear β-catenin expression linked to higher mortality in elderly patients.^306^ Similar mutations are also reported in melanoma.^307^ Interestingly, CTNNB1 and AXIN1 mutations are not typically observed in precancerous liver lesions but are associated with advanced tumor stages.^295,308,309^ Wnt pathway activation is predominantly seen in late-stage HCC, suggesting its involvement as a late event in liver carcinogenesis.^310^ CTNNB1 and AXIN1 mutations are correlated with distinct liver cancer subtypes, each presenting unique clinical and pathological characteristics. CTNNB1 mutations are more commonly associated with “non-proliferative” liver cancers, which include chromosomally stable tumors that retain differentiation markers and hepatocyte-like characteristics.^311^ Beyond the commonly studied mutations, the Wnt pathway also harbors mutations in other components, such as Fzd, LRP5/6, and TCF/LEF transcription factors.^312,313^ While Wnt pathway mutations are present in most cancer types, they exhibit significant tissue specificity. According to the TCGA database, CRC exhibits the highest frequency of APC mutations (67%) in sporadic tumors, followed by RNF43 (8%), CTNNB1 (6%), and AXIN2 (5%) mutations. Conversely, liver cancer preferentially acquires mutations in CTNNB1 (25%) and AXIN1 (8%), while pancreatic cancers often harbor RNF43 (6%) mutations, and adrenocortical cancers show mutations in ZNRF3 (20%) or CTNNB1 (15%).^314^ The impact of genetic alterations in the Wnt pathway on cancer development is likely more significant than current databases reflect, underscoring the need for further investigation into these mutations (Table 2).

**Table 2:** Mutated genes of Wnt signaling pathway

Cancer type	Mutated genes	Functional role	Refs.
Hepatocellular carcinoma	AXIN1	Immune evasion	^295^
	CTNNB1	Tumor tumorigenesis and progression	^302^
Advanced prostate cancer	AXIN1	Tumor tumorigenesis	^298^
Gastric cancer	AXIN1	/	^299^
	AXIN2	/	^299^
	RNF43	DNA damage response	^291^
Colorectal cancer	AXIN2	Tumor tumorigenesis	^300^
	RNF43	Tumor growth and recurrence	^288^
	APC	Tumor tumorigenesis	^280^
	CTNNB1	/	^306^
Pancreatic cancer	RNF43	/	^286^
Adrenocortical cancer	ZNRF3	Tumor recurrence	^293^
Acute myeloid leukemia	GSK-3β	Myelodysplastic	^301^
Medulloblastoma	CTNNB1	Prognosis	^305^
Melanoma	CTNNB1	Tumor progression	^307^

### Wnt signaling pathway in cancer stem cell biology

**Fig. 5:**
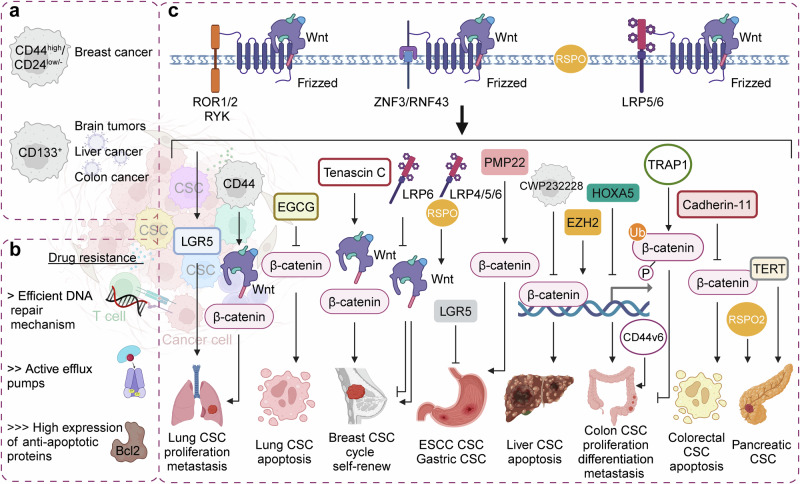
Wnt signaling pathway in cancer stem cells (CSCs). **a** Common CSC marker. **b** Primary causes of CSCs’ high drug resistance. **c** Potential role of the Wnt signaling pathway in CSC across various tumors. ROR1/2 receptor tyrosine kinase-like orphan receptor 1 and 2, RYK tyrosine kinases, ZNF3 zinc fingers 3, RNF43 RING finger protein 43, LRP lipoprotein receptor-related protein, LGR5 leucine-rich repeat-containing G protein-coupled receptor 5, EGCG epigallocatechin-3-gallate, CSC cancer stem cell, RSPO R-spondin. Image created with BioRender (https://biorender.com/)

Cancer stem cells (CSCs) are a subset of cancer cells endowed with stem cell-like properties, notably the ability to self-renew and differentiate into multiple cell types.^314^ These cells are embedded within tumor populations and are primarily responsible for cancer recurrence and metastasis. A defining feature of CSCs is their pronounced drug resistance, which frequently leads to chemotherapy failure.^315,316^ CSCs undergo symmetric or asymmetric division, facilitating self-renewal and maintaining the CSC population’s stability. Their extensive differentiation potential contributes to tumor heterogeneity by generating various cell types within the tumor microenvironment. Distinct surface markers have been identified for CSCs across different cancers; for instance, in BC, CSCs are characterized by CD44^high^/CD24^low/-^, while in brain tumors, liver cancer, and colon cancer, CD133^+^ cells serve as CSC markers.^317–320^ CSCs are integral to tumor initiation, progression, metastasis, drug resistance, and recurrence. They drive tumor growth through mechanisms of self-renewal and differentiation, forming the core population of tumor cells. Their high migratory and invasive capacities enable them to detach from the primary tumor, traverse the bloodstream or lymphatic system, and establish metastases in distant tissues. The remarkable resistance of CSCs to conventional chemotherapy and radiotherapy is attributable to several mechanisms, including efficient DNA repair, active drug efflux *via* ABC transporters, and the high expression of anti-apoptotic proteins such as Bcl-2 family members and apoptosis inhibitory factors.^321–323^ Multiple signaling pathways regulate CSC characteristics, with the Wnt, Notch, Hh, and PI3K/Akt/mTOR pathways being particularly prominent.^324^ This overview concentrates on the Wnt signaling pathway’s role in CSCs across various cancers. In some instances, the tumor microenvironment (TME) cells secrete cytokines, such as Wnt protein, BMP secretion inhibitor, and Delta, which activate signaling pathways essential for CSC self-renewal.^325^ Activation of the Wnt pathway transforms dormant CSCs into active ones through β-catenin, which drives cell cycle progression and upregulates cyclin D1 and MYC expression downstream.^326^ In cancers without identifiable genetic alterations in Wnt signaling, the knockdown of β-catenin may be linked to Wnt’s role in establishing and maintaining CSC populations.^327,328^ The Wnt signaling pathway is central to regulating differentiation balance in adult stem cells within various microenvironments, such as skin, hair follicles, breast, and intestines. Studies have underscored the Wnt pathway’s critical role in sustaining CSCs across different cancer types. The relationship between Wnt-driven stem cells and carcinogenesis is further substantiated by evidence linking Wnt signaling intensity to stem cell characteristics and colon CSCs behavior.^329–331^ Germ cell tumors, which closely mimic normal embryonic development, provide unique models for studying stem cells’ roles in tumorigenesis. Dysregulation of the Wnt pathway in these tissues leads to tumor proliferation. For instance, while CD133 deficiency does not impair human embryonic stem cells pluripotency or their differentiation into the three germ layers in vivo, it markedly diminishes cell proliferation. RNA-seq analysis indicates that CD133 deletion disrupts the regulation of p53, PI3K-Akt, AMPK, and Wnt signaling pathways. These pathway alterations are intricately linked to tumor proliferation and the evasion of apoptosis.^332^ Wnt signaling is essential for preserving the CD34^+^ CSC phenotype, with β-catenin loss leading to the depletion of CD34^+^ CSCs and complete tumor regression.^333^ In both mouse and human lung adenocarcinomas, two distinct cell subpopulations have been identified: one characterized by elevated Wnt signaling and the other forming a niche that supplies Wnt ligands. Wnt-responsive cells, marked by LGR5 expression, play a pivotal role in enhancing tumor proliferation. Although they represent a minor fraction of the tumor, these cells possess the capacity to generate diverse cell populations within the tumor. Evidence indicates that cancer cells with active Wnt signaling exhibit characteristics akin to normal tissue stem cells. Disruption of Wnt signaling, both genetically and pharmacologically, significantly hinders tumor progression in this context.^330^ For example, epigallocatechin gallate promotes apoptosis in lung mesenchymal stem cells by degrading β-catenin, while CD44 facilitates lung CSCs metastasis *via* the Wnt/β-catenin-FoxM1-Twist signaling pathway.^334,335^ In BC, the extracellular matrix protein tenascin C, commonly found in the stem cell niche, influences the cell cycle by enhancing Wnt signaling.^336^ Studies demonstrate that proto-oncogenes activate PKM2 to catalyze the final step of glycolysis through the Wnt pathway, essential for the growth of breast CSCs by upregulating β-catenin expression.^337–339^ Inhibiting Wnt signaling *via* LRP6 suppresses BC cell self-renewal and tumor seeding in vivo, while also inducing the re-expression of mammary epithelial differentiation markers and inhibiting EMT transcription factors SLUG and TWIST.^340^ The LGR4/5/6 receptors bind RSPO, thereby enhancing Wnt3a and activating Wnt signaling.^341,342^ LGR5 acts downstream of the Wnt pathway, where it inhibits the differentiation of esophageal squamous cell carcinoma stem cells.^343^ In gastric cancer, the capillary morphogenetic gene 2 increases nuclear β-catenin expression, regulating gastric CSC self-renewal and tumorigenicity.^344^ SMYD3 plays a key role in the epigenetic regulation of the Wnt signaling pathway, essential for ASCL2 activation and CSC maintenance.^345^ Additionally, PMP22 regulates gastric cancer cell self-renewal and chemotherapy resistance.^346^ CWP232228, an antagonist of β-catenin’s binding to nuclear TCF, induces apoptosis in liver CSCs.^347^ In colon cancer, high Wnt signaling is indicative of CSCs, with Wnt-high tumor cells located near stromal myofibroblasts that respond to secreted factors by activating β-catenin-dependent transcription.^331^ EZH2 enhances the expansion of colorectal CSC-like cells by modulating the p21cip1-Wnt/β-catenin pathway. HOXA5, on the other hand, inhibits stem cell properties by blocking the Wnt pathway in CRC. Retinoids have been shown to induce HOXA5-mediated tumor regression, offering a potential strategy for eliminating CSCs in colon cancer treatment.^348^ TRAP1, a member of the heat shock protein 90 chaperone family, inhibits colorectal CSC differentiation by regulating β-catenin ubiquitination and phosphorylation.^349^ Cadherin-11 functions as a tumor suppressor that promotes apoptosis by reducing active phosphorylated β-catenin levels and inducing apoptosis in colorectal CSCs, although it remains silent in ordinary tumors.^350^ CD44v6 serves as a marker of constitutive and reprogrammed CSCs that drive colon cancer metastasis. Tumor-associated cytokines, such as hepatocyte growth factor, osteopontin, and stromal-derived factor 1α, enhance CD44v6 expression in colorectal CSCs by activating the Wnt/β-catenin pathway, thereby facilitating migration and metastasis.^351^ In pancreatic cancer, varying levels of Wnt signaling correspond to distinct cancer cell characteristics. Cancer cells with high intrinsic Wnt signaling exhibit CSC properties, including increased tumor-initiating capacity and drug resistance, while those with low Wnt activity express differentiation markers. RSPO2 enhances Wnt signaling, conferring stem cell properties to susceptible pancreatic cancer cells. In prostate CSCs, TERT, an RNA-dependent DNA polymerase, forms a complex with β-catenin, thereby activating downstream targets of the Wnt pathway (Fig. 5).^352–354^

Extensive research has highlighted the significant role of non-coding RNAs and the Wnt signaling pathway in regulating the stemness of cancer cells.^105^ LncRNAs, which are RNA molecules exceeding 200 nucleotides and do not encode proteins, are pivotal in gene expression regulation, cell differentiation, development, and disease processes. In liver CSCs, lncTCF7 is notably upregulated and activates Wnt signaling by recruiting the SWI/SNF complex to the TCF7 promoter, thereby inducing TCF7 expression. This activation is critical for the self-renewal and tumorigenic potential of liver CSCs.^355^ Similarly, lnc-β-Catm stabilizes β-catenin in an EZH2-dependent manner, which is vital for maintaining the self-renewal capacity of hepatic CSCs.^356^ MiRNAs, typically 21–25 nucleotides in length, modulate gene expression by binding to the 3’UTR of target mRNAs. For example, miR-146a supports the symmetric division of colorectal CSCs by inhibiting Numb and stabilizing β-catenin.^357^ In glioma stem cells, temozolomide induces significant apoptosis by targeting the Wnt/β-catenin pathway, especially when combined with miR-125b.^358^ In BC, miR-142 recruits APC mRNA into the RNA-induced silencing complex, thereby inhibiting APC and activating the Wnt pathway, which subsequently upregulates miR-150 expression.^359^ Moreover, miR-582-3p targets the degradation of negative regulators of the Wnt pathway, such as AXIN2, DKK3, and SRP1, thus preserving the stemness features of non-small cell lung cancer (NSCLC) cells.^360^ MiR-1246 activates the Wnt signaling pathway by inhibiting AXIN2 and GSK3β, which in turn promotes cancer cell self-renewal, drug resistance, tumorigenicity, and metastasis.^361^ Similarly, miR-544a downregulates GSK3β, thereby sustaining the self-renewal ability of lung CSCs.^362^ Additionally, miR-483-5p enhances the growth, invasion, and self-renewal of gastric CSCs through the Wnt signaling pathway.^363^ CircRNAs, which are non-coding RNAs with a closed-loop structure, exhibit greater stability than linear RNAs. Several studies have demonstrated the influence of circRNAs on the Wnt pathway and their regulatory role in CSCs.^105^ For instance, circ-ABCC1 promotes CRC progression by activating the Wnt/β-catenin pathway, and exosomes derived from CD133^+^ cells carrying circ-ABCC1 enhance cell stemness and metastasis in CRC.^364^ Zhang et al. reported that circAGFG1 is upregulated in CRC cell lines; silencing circAGFG1 markedly inhibits cell proliferation, migration, invasion, and stemness while promoting apoptosis in CRC, primarily by regulating the Wnt/β-catenin pathway through CTNNB1.^365^ Additionally, exosomal circ_0030167 from bone marrow-derived mesenchymal stem cells (BM-MSCs) inhibits the invasion, migration, proliferation, and stem cell properties of pancreatic cancer cells by sponging miR-338-5p and targeting the Wif1/Wnt8/β-catenin axis.^366^

In conclusion, the critical importance of Wnt signal transduction in CSCs is increasingly recognized. Targeting the Wnt pathway presents a promising strategy for effectively regulating CSCs, offering potential avenues for innovative cancer treatments and preventive interventions.

### Wnt signaling pathway in tumor microenvironment

The tumor microenvironment (TME) is a critical factor in tumor progression, consisting of tumor cells, fibroblasts, keratinocytes, immune cells, and noncellular components. The interaction between tumors and the TME plays a vital role in promoting tumor cell proliferation, local invasion, and metastasis.^367,368^ Wnt signaling, a key pathway in tissue morphogenesis during embryonic development and repair, also significantly influences tumor initiation and progression through its interactions with the TME.^369,370^

The role of Wnt signaling in the immune system was first identified in T-cell development within the thymus, where β-catenin and TCF1 interaction, mediated by Wnt1 and Wnt4, enhances fetal thymocyte survival in vitro, highlighting its importance in thymocyte development.^371,372^ Research has further revealed that Wnt signaling regulates the immunogenicity of malignant cells and modulates immune responses, dynamically influencing the interactions between tumors and immune cells.^373^ In melanoma, mutated β-catenin overexpressed by tumor cells can be recognized by autologous cytotoxic T lymphocytes as tumor-associated antigens).^374^ However, Wnt pathway activation in malignant cells has been shown to suppress C-C motif chemokine ligand 4 (CCL4) secretion, impairing Batf3-dependent dendritic cell infiltration and activation.^375^ Regulatory DCs are pivotal in maintaining immunological tolerance by promoting the generation of regulatory T cells (Tregs) and inducing T cell unresponsiveness or apoptosis.^376^ Both canonical and non-canonical Wnt signaling in DCs increase IL-10 secretion, reduce IL-12 production, and elevate indoleamine 2,3-dioxygenase 1 levels, collectively fostering Treg generation, inhibiting CD8^+^ T cell function, and suppressing antitumor immune responses, particularly in melanoma.^377–380^ Furthermore, the deletion of Wnt co-receptors LRP5 and LRP6 in DCs may shift the balance toward promoting T cell differentiation while inhibiting Treg development, thereby influencing the immune landscape and potentially affecting tumor progression.^381^ Several studies have demonstrated a direct link between β-catenin expression and the infiltration, survival, and functionality of Tregs.^382,383^ Loosdregt et al. found that Wnt signaling activation under inflammatory conditions disrupted Foxp3 transcriptional activity, reducing Treg function and promoting a more robust immune response.^382^ Conversely, Keerthivasan and colleagues reported that Wnt signaling activation in Tregs promoted colitis-associated cancer, underscoring the pathway’s context-dependent effects.^384^ It is well-established that inhibiting Wnt signaling enhances effector T cell infiltration and activation. For example, β-catenin inhibition promoted T cell infiltration, transforming the colorectal TME into a T-cell inflamed phenotype, likely improving the efficacy of various immunotherapies in treating CRC.^385^ The loss of the tumor suppressor PTEN, however, has been shown to impair T cell function through the increased expression of DKK2, independent of LRP6 and β-catenin signaling.^386^ Wnt signaling also plays a pivotal role in macrophage phenotype regulation. Wnt5a enhances the invasiveness induced by macrophages, promoting tumor cell migration.^387,388^ It inhibits the differentiation of macrophages into the pro-inflammatory M1 type, driving the production of immunosuppressive cytokines such as TGF-β and IL-10, which fosters an M2 macrophage-like phenotype.^389^ Kaler et al.^390^ demonstrated that tumor cells stimulate macrophages to release IL-1β, which leads to GSK3β phosphorylation, stabilizing β-catenin, enhancing TCF-dependent gene activation, and promoting Wnt target gene expression in tumor cells. Furthermore, oncogenic β-catenin mutations in CRC cells, alongside Wnt signaling activation, trigger IL-1β production in macrophages *via* Snail.^391^

In non-immune cells within the TME, Wnt signaling also significantly impacts tumor progression. CRC cells upregulate the tumor suppressor gene, insulin-like growth factor-binding protein 7 (IGFBP7), in fibroblasts *via* the coordinated regulation of TGF-β and Wnt signaling through Smad2/3-Dvl2/3 interactions.^392^ Tumor-associated fibroblasts release Wnt2 protein, enhancing tumorigenesis and aggressiveness.^393^ Additionally, cysteine-rich 61, an ECM protein, is identified as a β-catenin target gene, facilitating HCC progression by modulating hepatic stellate cells.^394^ Activation of the β-catenin/MMP-7 signaling pathway promotes EMT, enhances cellular migration, and drives the malignant progression of cancer cells.^395^

Tumor-derived Wnt signaling plays a pivotal role in facilitating immune evasion and establishing an immunosuppressive microenvironment through various mechanisms. These include the upregulation of immune checkpoint molecules, recruitment and polarization of immunosuppressive macrophages, and ECM modification.^396,397^ Castagnoli et al. identified significant crosstalk between Wnt activation and programmed death-ligand 1 (PD-L1) expression in triple-negative breast cancer (TNBC), highlighting the role of β-catenin in regulating PD-L1 levels.^398^ Specifically, the binding of the β-catenin/TCF/LEF complex to the promoter region of the CD274 gene drives PD-L1 expression on tumor cells, underscoring the importance of Wnt signaling in immune evasion.^399,400^ Harnessing the potential of Wnt signaling in cancer immunotherapy could lead to more effective and durable treatments. Beyond direct regulation of signaling pathways, the interaction between β-catenin and prostaglandin E2 (PGE2) or liver kinase B1 (LKB1) in modulating PD-L1 expression has garnered significant attention.^401,402^ M2 tumor-associated macrophages secrete PGE2, promoting PD-L1 expression, while LKB1 contributes to β-catenin activation, which, in turn, regulates PD-L1 activity.^401,402^ The silencing or deletion of LKB1 elevates PD-L1 levels, increasing resistance to anti-PD-L1 therapies.^402–404^ Moreover, PD-L1 plays a critical role in metabolic changes within both cancerous and immune cells.^396^ Inhibition of PD-L1 can reduce glycolysis in cancer cells, thereby increasing glucose availability in the TME and enhancing T cell function.^405^ These metabolic effects offer additional opportunities for improving immunotherapy outcomes.

**Fig. 6:**
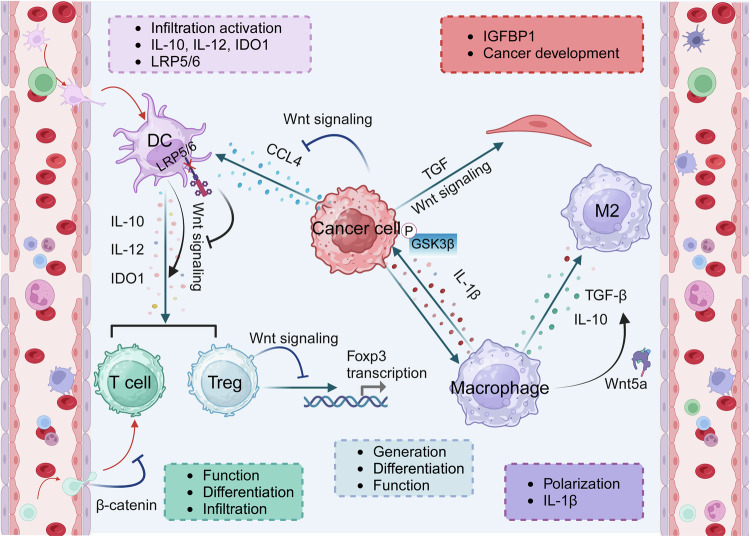
The Wnt signaling pathway plays a crucial role in modulating the immune microenvironment of tumors. Wnt activation suppresses the secretion of CCL4 and reduces the infiltration and activation of dendritic cells in tumor cells. Regulatory destruction complexes play a vital role in maintaining immunological tolerance by aiding in the development of regulatory T cells and fostering T cell unresponsiveness or apoptosis. Furthermore, Wnt signaling plays a vital role in modulating the equilibrium among various macrophage phenotypes. DC dendritic cell, IDO1 Indoleamine 2,3-dioxygenase 1, TGF Transforming growth factor, CCL4 C-C motif chemokine ligand 4, IL Interleukin. Image created with BioRender (https://biorender.com/)

In conclusion, a deeper understanding of the molecular mechanisms governing Wnt-mediated immune modulation and tumor-immune interactions is essential for addressing the challenges in tumor immunotherapy. Novel insights into these pathways may inform therapeutic strategies that can effectively reverse the immunosuppressive microenvironment, potentially leading to more successful cancer treatments (Fig. 6).

## Wnt signaling pathway in human diseases

### Cardiovascular disease

#### Atherosclerosis

Atherosclerosis is a major driver of various cardiovascular diseases, including ischemia, myocardial infarction, and stroke.^406^ Wnt signaling is involved in all stages of atherosclerosis, from endothelial dysfunction to plaque formation.^407,408^ A previous study revealed that Wnt4 mediates the effects of circUSP36 on endothelial cell behavior.^409^ Mechanistically, circUSP36 promotes Wnt4 expression by binding competitively to miR-637, which exacerbates endothelial dysfunction in atherosclerosis. Impaired cholesterol transport is also central to atherosclerotic lesion development, and growing evidence suggests that Wnt5a plays a key role in regulating cholesterol homeostasis.^410–414^ Awan and colleagues found that Wnt5a downregulates the PI3K/Akt/mTORC1 pathway in human VSMCs, facilitating lysosomal cholesterol egress to the endoplasmic reticulum, thereby offering protection against atherosclerosis.^415^ However, another study showed that Wnt5a overexpression leads to cholesterol accumulation and inflammatory responses in VSMCs through activation of the ROR2/ABCA1 axis and NF-κB translocation to the nucleus.^416^ These findings suggest that Wnt5a’s regulatory role in cholesterol homeostasis is highly complex and requires further investigation.

Pathological vascular remodeling is another hallmark of atherosclerosis.^417^ Recent studies suggest that β-catenin activates the promoter of sphingosine-1-phosphate receptor 1 (S1PR1) *via* its C-terminal domain, inducing S1PR1 protein expression in smooth muscle cells and promoting vascular remodeling and atherosclerosis.^418^ In recent years, complications from atherosclerosis have become increasingly fatal, making the regression of atherosclerosis a critical clinical goal.^419,420^ Weinstock et al. observed increased Wnt signaling in macrophages during plaque regression.^421^ Wnt3a activates STAT3, inducing PGE2 production, which enhances macrophage responses to IL-4 and promotes the resolution of atherosclerosis. Overall, these findings emphasize the pivotal role of Wnt signaling in regulating endothelial cell function, cholesterol homeostasis, vascular remodeling, and inflammation, offering potential new avenues for treating atherosclerosis.

#### Arrhythmogenesis

Arrhythmogenesis, characterized by abnormal electrical pulse generation or conduction, has an annual incidence of approximately 0.5%.^422^ Research has shown that Wnt signaling regulates the expression of connexin43, a key ion-conducting hemichannel that connects cardiomyocytes at gap junctions, thus contributing to cardiac electrical stability and influencing arrhythmogenesis.^423^ Follow-up studies have revealed that Wnt signaling regulates genes involved in cardiac conduction in a chamber-specific manner, affecting both the right and left ventricles.^424^ In particular, YRPW motif protein 2, a transcriptional repressor linked to Brugada syndrome, is a direct target of Wnt signaling in the right ventricle, increasing susceptibility to right ventricular tachycardia in adults.

Atrial fibrillation (AF) is the most common cardiac arrhythmia and is associated with life-threatening conditions such as stroke and heart failure, placing a significant strain on healthcare systems.^425^ Numerous studies have demonstrated marked dysregulation of Wnt signaling components during AF, suggesting that modulating this pathway could influence the progression of the condition.^426–428^ For example, Tan et al. found that the lncRNA HOTAIR enhances Wnt5a stability by binding to PTBP1, which promotes myocardial fibrosis in AF.^429^ Additionally, inhibiting Fzd8 to deactivate the Wnt5a pathway alleviates AF in rat models.^430^ Moreover, modulation of the Wnt-β-catenin-induced PI3K/Akt pathway, which activates protein C, has shown beneficial effects in mice with thromboembolic-induced AF.^431^ These findings suggest that further research into the role of Wnt signaling in AF could lead to the development of novel targeted therapies for managing the progression of AF and other arrhythmias.

#### Arrhythmogenic cardiomyopathy

Arrhythmogenic cardiomyopathy (ACM) is a hereditary myocardial disease characterized by cardiac arrhythmias, progressive heart failure, and sudden cardiac death.^432,433^ Research has shown that the canonical Wnt pathway is inhibited in human ACM without evident heart failure.^434^ In a novel transgenic mouse model of ACM, featuring cardiomyocyte-specific overexpression of human desmoglein-2, downregulation of the Wnt/β-catenin signaling pathway was also observed.^435^ Khudiakov and colleagues further identified that modulating the activity of the GSK3β-Wnt/β-catenin signaling can influence the function of cardiac sodium channels, highlighting its potential involvement in ACM pathogenesis.^436^ Arrhythmogenic right ventricular cardiomyopathy (ARVC), a major cause of sudden death in young adults, is pathologically characterized by the replacement of the myocardium with fibroadipose tissue.^437,438^ While the genetic basis of ARVC is not fully understood, mutations in genes encoding desmosomal proteins such as desmoplakin, plakophilin-2, desmoglein-2, and desmocollin-2 have been identified as causes of the common autosomal dominant form of ARVC.^439,440^ Studies have demonstrated that the inhibition of canonical Wnt/β-catenin signaling, mediated by TCF/LEF1 transcription factors alongside nuclear plakoglobin, can replicate the human ARVC phenotype.^441^ β-catenin plays a central role in the degradation of cardiomyocytes and the formation of adipose tissue in ARVC.^442,443^ Dysregulation or absence of Wnt/β-catenin signaling, inhibition of Dvl, and elevated levels of GSK3β and CK1 are key cytotoxic events that trigger apoptosis.^444^ Moreover, Wnt/Ca^2+^ signaling regulates the expression of Bcl2 and inhibits mitochondrial apoptosis by initiating endoplasmic reticulum stress, contributing to the pathogenesis of ARVC. These insights into Wnt signaling provide a better understanding of the molecular mechanisms underlying ARVC and offer potential therapeutic targets for managing the disease.

#### Myocardial infarction

Myocardial infarction (MI), defined as the death of myocardial cells due to prolonged ischemia, poses a significant life-threatening risk and remains one of the leading causes of global mortality.^445,446^ Studies have shown that canonical Wnt signaling is activated in the infarcted area of experimental MI, contributing to endothelial-to-mesenchymal transition (EndMT).^447^ Additionally, non-canonical Wnt signaling in the cardiac microenvironment post-MI promotes the activation of inflammatory monocytes, influencing the prognosis of the condition.^448^ Another study highlighted that Wnt signaling increases in the bone marrow after acute MI, driving the proliferation of hematopoietic stem cells, which play a role in tissue repair.^449^ Various Wnt ligands affect MI progression through distinct mechanisms. For example, Goliasch et al. found that premature MI was associated with reduced serum Wnt1 levels.^450^ In contrast, upregulated Wnt2 and Wnt4 post-MI activate the β-catenin/NF-κB signaling pathway *via* the cooperative action of Fzd4/2 and LRP6, promoting myocardial fibrosis and worsening cardiac dysfunction.^260^ On the other hand, Wnt3a has been shown to improve myocardial function in elderly patients with acute MI by reducing mitochondrial oxidative stress induced by Cys-C/ROS signaling.^451^ Moreover, non-canonical Wnt5a-PCP signaling facilitates wound repair and prevents cardiac rupture after MI by mediating TGFβ1-Smad2/3-dependent CTHRC1 activation.^452^

Cardiac Wnt/Fzd signaling is activated following myocardial infarction. Thus, blocking Wnt/Fzd signaling is a potential therapeutic strategy to enhance cardiac repair after MI.^453,454^ sFRP bind directly to Wnt ligands, preventing Wnt/Fzd interactions and inhibiting the Wnt signaling cascade.^455,456^ Alvandi and colleagues demonstrated that sFRP3 can block Wnt/β-catenin and forkhead box M1 (FOXM1), protecting the mitral valve endothelium from EndMT following MI.^457^ Similarly, sFRP5 was shown to inhibit Wnt5a/JNK signaling, promoting proliferation, migration, and angiogenesis of human umbilical vein endothelial cells and mitigating myocardial injury in diabetic MI mice.^458^ In addition to sFRPs, traditional Chinese medicines and natural herb extracts, such as Linggui Zhugan decoction, Liensinine, Berberine, and Huoxin pill, have demonstrated myocardial protective effects by inhibiting the Wnt/β-catenin signaling following MI.^459–462^ In summary, a deeper understanding of the molecular and cellular mechanisms involved in Wnt signaling during cardiac repair is critical for improving clinical outcomes for patients with myocardial infarction.

### Neurodegenerative diseases

#### Parkinson’s disease

Parkinson’s disease (PD) is a neurodegenerative disorder characterized by the selective loss of dopaminergic neurons in the substantia nigra pars compacta.^463,464^ Mutations in the leucine-rich repeat kinase 2 (LRRK2) gene, particularly the G2019S variant, represent the most common genetic cause of PD.^465^ In a PD LRRK2 G2019S knock-in model, dysregulated Wnt signaling has been observed.^466^ Wnt signaling also acts a vital role in the activation of nuclear receptor-related 1 (Nurr1), a transcription factor critical for the development, differentiation, and maintenance of midbrain dopaminergic neurons.^467^ This pathway is integral to various aspects of neural development, including axon extension and synapse formation, with increasing evidence indicating that Wnt signaling enhances neuroprotection and self-repair in PD.^468–475^ Studies have revealed that activation of Wnt/β-catenin signaling through the downregulation of GSK3β promotes neuroprotection and repair.^476^ In contrast, Haynes et al. found that inhibition of Wnt/β-catenin signaling upregulates the transcriptional repressor NR0B1, impacting the dopaminergic neuronal phenotype.^477^ Additionally, activation of dopamine D1 receptors has been found to positively regulate the Wnt/β-catenin signaling cascade, ameliorating hippocampal nerve damage in adult PD rats.^478^ Axin-2, a negative regulator of the Wnt/β-catenin signaling, plays a key function in modulating this pathway. Knocking down Axin-2 upregulates Wnt/β-catenin signaling, which protects mitochondrial function, promotes dopaminergic neurogenesis, and improves behavioral function in PD rats.^479^ These findings underscore the critical role of Wnt signaling in dopaminergic neurogenesis and point to its potential as a therapeutic target for PD. In conclusion, Wnt signaling is essential for the regeneration and protection of dopaminergic neurons. Continued research into Wnt signaling mechanisms may lead to breakthroughs in endogenous brain repair and the development of new therapeutic strategies for PD.

#### Alzheimer’s disease

Alzheimer’s disease (AD) is a neurodegenerative disorder defined by the accumulation of amyloid-β and phosphorylated tau proteins, leading to progressive, age-related cognitive decline.^480–482^ Extensive research has identified dysregulated Wnt signaling as a contributing factor in AD pathogenesis.^483–488^ For instance, the deletion of the neuronal LRP6 gene leads to the downregulation of Wnt signaling, causing synaptic dysfunction and amyloid pathology.^489,490^ A recent study highlighted that the downregulation of Wnt1, PORCN, and RSPO2 in the brains of patients with AD synergistically inhibits the Wnt/β-catenin signaling pathway.^491^ Moreover, increased GSK3β kinase activity in the prefrontal cortex has been linked to AD pathology.^492,493^ Given the central role of Wnt signaling in AD, it presents a compelling target for therapeutic intervention.^494–498^ Xu et al. found that upregulating Wnt2a improves mitochondrial function and offers neuroprotection in AD models.^499^ Yoon et al. demonstrated that inhibiting CXXC5 function restores Wnt/β-catenin signaling, alleviating neuronal inflammation and cognitive deficits.^500^ Further preclinical studies suggest that enhancing Wnt/β-catenin signaling, through methods such as using the MST1 selective inhibitor Xmu-mp-1, downregulating the Wnt antagonist Dickkopf-3, or increasing APOE3 Christchurch expression, can mitigate AD pathology.^501–503^ Collectively, these findings provide a foundation for developing innovative therapeutic strategies for AD within the framework of Wnt signaling.

#### Huntington’s disease

Huntington’s disease (HD) is an autosomal dominant neurodegenerative disorder caused by the abnormal expansion of trinucleotide repeats in the huntingtin gene.^504^ First described in the 19th century, HD is featured by progressive cellular degeneration in the striatum and cerebral cortex, resulting in chorea, cognitive impairment, and psychiatric disturbances.^505,506^ While relatively few studies have examined Wnt signaling in HD, Lim et al. unraveled that Wnt/β-catenin signaling is activated in human HD brain tissue, and Wnt inhibition was shown to prevent angiogenic defects in vitro.^507,508^ Charlene et al. demonstrated that targeted inhibition of Wnt signaling eliminates the neural stem cell population in HD neuronal cultures, suggesting that Wnt signaling could be a promising therapeutic target for HD.^509^ Additionally, in 3-Nitropropionic acid-induced HD model rats, Wnt/β-catenin signaling mediates the neuroprotective effects of lercanidipine against neurotoxicity.^510^ These encouraging findings suggest that targeting the Wnt pathway could offer a promising therapeutic approach to slow HD progression. However, further studies are needed to elucidate the precise mechanisms by which the Wnt pathway influences HD pathology.

#### Amyotrophic lateral sclerosis

Amyotrophic lateral sclerosis (ALS) is a fatal neurodegenerative disorder characterized by the progressive loss of motor neurons in the brain and spinal cord, leading to muscle atrophy and eventual paralysis.^511^ Emerging evidence suggests that Wnt signaling plays a role in the neurodegenerative processes associated with ALS.^512–515^ In transgenic ALS mouse models, neurodegeneration was found to increase the expression of Wnt1, Wnt2, Wnt4, Wnt5a, Wnt7a, Fzd2, and Fzd5 in the spinal cord, with co-localization observed between Wnt1 and Fzd1, as well as Wnt5a and Fzd2, indicating activated Wnt signaling.^516–521^ However, other studies suggest a protective role of Wnt signaling; for example, Wnt5a was found to enhance cell viability and promote axon outgrowth *via* the non-canonical Wnt/Ca^2+^ signaling pathway, potentially protecting motor neurons in ALS.^522^ Pathological changes in skeletal muscle and neuromuscular junctions are observed early in ALS. Kwan et al. reported upregulation of sFRPs, Wnt antagonists, and β-catenin in ALS muscle myofibers, reflecting the complex molecular responses to muscle denervation.^523^ Animal models also indicate that blood-brain barrier disruption may precede neurodegeneration in ALS. In a novel ALS patient-derived brain microvascular endothelial cell model (TARDBP^N345K/+^), downregulation of Wnt/β-catenin signaling was identified, suggesting that Wnt signaling-mediated vascular barrier dysfunction may contribute to ALS pathogenesis.^524^

### Metabolic disorders

#### Type 2 diabetes mellitus

Type 2 diabetes mellitus (T2DM) is a prevalent chronic condition associated with significant morbidity and mortality, posing a substantial public health challenge.^525^ A genetic epidemiological study in the Han Chinese population identified potential gene-gene interactions between T2DM and key components of the canonical Wnt signaling pathway, including LRP5, TCF7L2, and the downstream glucagon gene, suggesting that this pathway may influence T2DM risk.^526^ Additional research demonstrated that overexpression of the Wnt5b gene in preadipocytes promotes adipogenesis, potentially contributing to T2DM pathogenesis by modulating adipocyte function.^527^ Since the Wnt pathway regulates lipid metabolism, insulin signaling, and glucose homeostasis, it presents a promising target for T2DM treatment and its associated complications.^528–532^ T2DM also impairs bone microarchitecture and quality, heightening fracture risk. Giulia et al. observed that canonical Wnt signaling in bone is suppressed in T2DM, correlating with elevated advanced glycation end product levels and reduced bone strength.^533^ Other studies have similarly shown that activation of Wnt3a/β-catenin signaling may exacerbate bone fragility in T2DM.^534–536^ Furthermore, bone morphogenetic protein 2 promotes osteogenesis in T2DM bone marrow stromal cells by activating Wnt/β-catenin signaling and inhibiting GSK3β.^537^ T2DM is also an independent risk factor for Alzheimer’s disease, with research indicating that Wnt/β-catenin signaling modulates brain insulin regulation, mitigating cognitive decline in T2DM.^538,539^ In diabetic nephropathy, the Nrf2/Wnt/β-catenin pathway has been implicated in zinc-mediated protection against T2DM-related renal cell apoptosis.^540^ In conclusion, further investigation into the mechanistic links between the Wnt signaling pathway and T2DM complications is essential for optimizing disease management.

#### Obesity

Obesity is characterized by excessive fat accumulation and chronic low-grade inflammation, with its global prevalence rising, making it a major public health concern.^541^ Wnt signaling plays a critical role in maintaining cellular homeostasis and energy balance, spanning from the hypothalamus to metabolic organs.^542,543^ Jonas et al. demonstrated that obesity impairs hypothalamic Wnt signaling, which can be restored with leptin treatment.^544^ Another study highlighted that Wnt5a-driven non-canonical Wnt signaling contributes to obesity-induced insulin resistance and metabolic dysfunction by amplifying inflammation in adipose tissue.^545^ Moreover, Wnt5a, secreted from adipose tissue in obesity, triggers redox-dependent migration of VSMCs through activation of the Fzd2/USP17/RAC1 axis and increased NADPH oxidase activity.^546^ These insights suggest that modulating Wnt signaling pathways may offer novel therapeutic approaches for managing obesity. In mouse models of obesity-induced cognitive impairment, Wnt/β-catenin signaling mediates the regulation of FABP4, reducing neuroinflammation and cognitive decline.^547^ Gao et al. found that activation of canonical Wnt signaling enables embelin to suppress adipogenesis and improve glucose tolerance impaired by obesity.^548^ Additionally, Yue et al. identified that DHA-enriched phosphatidylcholine mitigates obesity-related osteoporosis by upregulating the Wnt/β-catenin pathway.^549^ Collectively, these findings provide promising avenues for addressing obesity and its complications.

#### Non-alcoholic fatty liver disease

Nonalcoholic fatty liver disease (NAFLD), a systemic metabolic disorder often linked to dyslipidemia, insulin resistance, and inflammation, has become a growing public health issue alongside the obesity epidemic, now affecting approximately 25% of the global population.^550,551^ Wnt signaling is essential for liver development, regeneration, metabolism, and detoxification, maintaining hepatic homeostasis.^552–555^ Previous studies showed that LRP6^mut/mut^ mice develop steatohepatitis and steatofibrosis through non-canonical Wnt signaling activation, while administration of Wnt3a to these mice can reverse liver abnormalities.^556^ Additional research revealed that the LRP6 genotype modulates individual susceptibility to NAFLD *via* the Wnt/β-catenin-Cyp2e1 signaling axis.^557^ Furthermore, inhibiting miR-21 expression may alleviate NAFLD by targeting LRP6 and activating the Wnt/β-catenin pathway.^558^ Gut-vascular barrier dysfunction has been recognized as a precursor to NAFLD. Ke et al. discovered that Wnt/β-catenin signaling activation protects the gut-vascular barrier, preventing E. coli NF73-1 translocation to the liver and reducing high-fat diet-induced NAFLD.^559^ Macrophage activation plays a pivotal role in advancing liver injury, and in pediatric NAFLD, Guido et al. demonstrated that macrophage modulation drives hepatic progenitor cell responses through Wnt3a production.^560^ The liver’s capacity for repair and regeneration is notably robust, and Li et al. found that Wnt/β-catenin signaling regulates Sirt1, promoting liver regeneration in NAFLD, suggesting potential therapeutic strategies for the disease.^561^

### Autoimmune diseases

#### Inflammatory bowel disease

Inflammatory bowel disease (IBD) is a chronic, non-specific inflammatory disorder of the intestine, arising from a dysregulated mucosal immune response to intestinal microbes in genetically predisposed individuals.^562^ The two main forms of IBD, Crohn’s disease (CD) and ulcerative colitis (UC), collectively impact around 10 million people worldwide.^563,564^ Extensive research has revealed that Wnt signaling is dysregulated in IBD and may contribute to its pathogenesis.^565–567^ Quandt et al. demonstrated that activated Wnt/β-catenin signaling in Tregs induces epigenetic reprogramming, altering the expression of proinflammatory genes co-regulated by TCF1 and Foxp3, thereby promoting the progression of IBD.^568^ Lan et al. found that macrophage-derived Wnt2b might be involved in IBD-related colon inflammation through competitive binding of IκB kinase-interacting protein, activation of the NF-κB pathway, and increased expression of downstream inflammatory mediators.^569^ In ileal CD, Ando et al. observed diminished activity of the PLC-β3-mediated Wnt/β-catenin pathway.^570^ Complications such as fibrosis and fistula formation are common in CD.^571^ Dolores et al. identified that Wnt2b induces EMT in vitro by activating Fzd4, correlating with intestinal penetration in patients with CD.^572^ In UC, chronicity is associated with increased M2 macrophages, which activate the Wnt signaling pathway in epithelial cells while inhibiting intestinal epithelial cell differentiation.^573^ Conversely, Kazuhiko et al. found a negative correlation between Wnt5a expression and the levels of TNF-α and IL-8 in the colonic mucosa of patients with UC, suggesting that Wnt5a may play an anti-inflammatory role in UC.^574^ These studies suggest that Wnt signaling can either promote or inhibit IBD progression, depending on the context. The mechanisms underlying these contrasting roles remain uncertain, and further research is needed to elucidate the specific regulatory functions of Wnt signaling in CD and UC.

#### Systemic lupus erythematosus

Systemic lupus erythematosus (SLE) is a highly heritable autoimmune disorder characterized by the loss of self-tolerance, leading to the formation of nuclear autoantigens and immune complexes, affecting various organs, including the kidneys, skin, and nervous system.^575^ A whole-genome sequencing study on a Chinese family identified a rare missense variant of Wnt16 associated with SLE. Unlike the wild-type, this Wnt16 variant failed to activate canonical Wnt/β-catenin signaling, underscoring the importance of Wnt signaling in maintaining immune homeostasis.^576^ Another study suggested that Wnt5a could serve as a non-invasive biomarker for assessing disease activity and the severity of skin involvement in patients with SLE.^577^ Furthermore, activated Wnt5a was shown to modulate the LINC00176/WIF1 signaling axis, promoting CD4^+^ T cell proliferation and adhesion in SLE.^578^ Additionally, Wnt/β-catenin signaling is thought to influence the senescence of BM-MSCs in SLE by activating the p53/p21 pathway, offering insights into improving BM-MSC transplantation outcomes in patients with SLE.^579^ Lupus nephritis, the most common organ manifestation of SLE, has been linked to abnormal activation of Wnt/β-catenin signaling, as reported by Wang et al.^580,581^ Activated Wnt/β-catenin signaling was found to mediate chemokine CX3CL1 to promote EMT and contribute to tubulointerstitial lesions in lupus nephritis.^582^ Collectively, these findings highlight the critical role of Wnt signaling in SLE pathogenesis and suggest new avenues for therapeutic interventions.

#### Rheumatoid arthritis

Rheumatoid arthritis (RA) is a chronic autoimmune inflammatory joint disorder marked by synovial hyperplasia, inflammation, and progressive cartilage loss, leading to joint destruction and, in severe cases, permanent disability.^583^ Wnt signaling has been identified as a key regulator of osteoblast differentiation and joint formation, with significant implications for synovial inflammation and bone remodeling.^584–586^ Li et al. discovered that Wnt/β-catenin signaling mediates IL-35-induced osteoblast differentiation in response to TNF-α activation in RA.^587^ In hTNF^tg/+^ transgenic mice, the depletion of Wnt9a aggravated TNF-driven inflammation, exacerbating pannus formation and joint destruction.^588^ Fibroblast-like synoviocytes (FLS), the primary contributors to pannus formation, play a critical role in RA progression. Dorra and colleagues found that Wnt5a enhances proinflammatory cytokine expression in FLS, inducing synovial dysfunction.^589^ In the acidic environment of RA synovial fluid, Wnt/β-catenin/c-Myc signaling, activated by acid-sensing ion channel 1a, promotes FLS proliferation.^590^ Moreover, the Wnt/β-catenin pathway facilitates RSPO2 to drive the invasive phenotype of RA synovial fibroblasts, disrupting chondrocyte homeostasis and worsening disease outcomes.^591^ Recent studies suggest that targeting Wnt signaling could mitigate RA severity, providing potential therapeutic insights.^592–595^ However, the precise role of Wnt signaling in RA remains to be fully elucidated, and further experimental research is needed to establish a robust theoretical framework for its clinical translation.

#### Multiple sclerosis

Multiple sclerosis (MS) is an autoimmune-mediated demyelinating disorder of the central nervous system, leading to neurological disability and a diminished quality of life.^596^ While early research suggested that canonical Wnt signaling negatively affects oligodendrocyte development and myelination, subsequent findings have demonstrated its role in promoting remyelination.^597^ In a cuprizone-induced mouse model of MS, activated Wnt/β-catenin signaling was found to mediate C1q, inhibiting the differentiation of oligodendrocyte progenitor cells and thereby worsening demyelination.^598^ In contrast, two downstream effectors of Wnt/β-catenin signaling, the PI3K/Akt pathway and TCF7L2, were upregulated, contributing to successful remyelination.^599^ These observations indicate that Wnt signaling exerts a complex regulatory influence on myelination processes. Blood-brain barrier dysfunction is an early hallmark of MS, facilitating immune cell infiltration and directly damaging the central nervous system.^600^ Studies have shown that activation of Wnt/β-catenin signaling enhances Claudin-1 expression, preserving blood-brain barrier integrity and limiting immune cell infiltration.^601,602^ Since relapse is a core feature of MS and a frequent complication in clinical settings, preventing recurrence is essential for improving patient outcomes.^603,604^ A large population-based study identified a positive correlation between variants of the Wnt9b gene and increased relapse risk in MS.^605^ Thus, further exploration of the role of Wnt signaling in MS disease activity is essential to unlock new therapeutic strategies.

### Cancer

#### Colorectal cancer

The Wnt signaling pathway takes a pivotal part in regulating cell growth, differentiation, apoptosis, and self-renewal, with its dysregulation being closely linked to the initiation and progression of various cancers. In CRC, mutations in the APC gene are frequently observed and are significant contributors to the development of familial adenomatous polyposis and its progression to CRC.^22,606^ Furthermore, aberrant activation of the Wnt/β-catenin pathway is associated with metastasis, recurrence, and resistance to anti-cancer therapies. Dvl3 is notably overexpressed in CRC tissues, promoting EMT and cancer stem-like cell (CSLC) properties through activation of the Wnt/β-catenin/c-Myc/SOX2 axis.^607^ In oxaliplatin-resistant CRC cell lines, a positive feedback loop between overactivation of Wnt/β-catenin signaling and IMPDH2 was identified, which inhibits caspase-dependent apoptosis and fosters drug resistance.^608^ CD45 also mediates the abnormal activation of Wnt signaling by stabilizing β-catenin, thereby enhancing both stemness and resistance to chemoradiation in CRC cells.^609^ A 2023 study revealed that NLRP12 inhibits GSK3β phosphorylation by interacting with STK38, leading to β-catenin degradation and suggesting new therapeutic possibilities for CRC.^610^ Additionally, the interplay between lncRNA and the Wnt pathway is significantly involved in CRC pathogenesis. Hypoxia-induced lncRNA STEAP3-AS1 activates Wnt/β-catenin signaling by inhibiting GSK3β *via* YTHDF2 and STEAP3, promoting CRC progression.^611^ Another study identified lncRNA RP11-417E7.1 as a driver of M2 macrophage polarization, fostering a pro-metastatic environment by activating the Wnt/β-catenin pathway in CRC.^612^ Moreover, m6A-modified BACE1-AS activates Wnt signaling in a TUFT1-dependent manner, promoting CSLC traits and liver metastasis in CRC.^154^

#### Liver cancer

In HCC, Wnt/β-catenin overactivation is detected in approximately 95% of cases, largely driven by gain-of-function mutations in the CTNNB1 gene.^613^ This aberrant signaling may be influenced by genetic factors, epigenetic changes (such as WIF-1 gene promoter hypermethylation), and viral infections. The heterogeneity of immunophenotypes in Wnt/β-catenin-mutated HCC is reportedly shaped by the activation of downstream transcription factors HNF4A and FOXM1.^614^ A recent study identified WNTinib, a multi-kinase inhibitor that selectively antagonizes CTNNB1-mutant HCC through the KIT/MAPK/EZH2 axis.^615^ In addition to mutations in classical Wnt components such as CTNNB1, APC, AXIN1, or AXIN2, elevated expression of non-canonical Wnt components like Wnt5a and ROR2 correlates with tumor differentiation in HCC.^616,617^ A 2023 study identified a Wnt/TGFB subclass of HCC characterized by cancer-specific ECM deposits, associated with poor patient outcomes.^618^ Additionally, abnormal SUMOylation of RNF146 promotes its interaction with Axin, accelerating Axin degradation and thereby enhancing Wnt/β-catenin signal transduction, contributing to HCC progression.^619^ GREB1, a specific Wnt target gene identified by Shinji Matsumoto and colleagues, acts as a Wnt mediator in cooperation with HNF4α and FOXA2, driving HCC proliferation.^620^ Another study found that ZMIZ2 enhances the malignant phenotype of HCC by interacting with LEF1 and activating the Wnt/β-catenin pathway.^621^ Wnt/β-catenin signaling is also critical in maintaining liver CSLC characteristics, with N6-methyladenosine methylation-mediated upregulation of Fzd10 contributing to lenvatinib resistance in HCC.^622^

#### Lung cancer

Aberrant activation of the Wnt pathway is closely linked to various biological processes in lung cancer, including cell proliferation, migration, invasion, and apoptosis. Smoking, a major risk factor for lung cancer, has been shown to induce the overexpression of key genes in the Wnt/β-catenin pathway, such as Wnt3, DLV3, AXIN, and β-catenin, in bronchial epithelial cells.^623^ NSCLC, the most common type of lung cancer, accounts for 80–85% of cases and remains a leading cause of cancer-related mortality globally. Wnt7a, a non-canonical Wnt ligand, has been identified as a tumor suppressor in NSCLC, but it is frequently downregulated.^624^ A 2022 study highlighted Wnt5a as a ligand for the non-canonical Wnt pathway, selectively upregulating RHOA to drive tumorigenesis and cell proliferation in SCLC.^625^ Another non-canonical ligand, Wnt5b, was found to bind to Fzd3 in NSCLC cells, recruiting Dvl3 for membrane phosphorylation, thereby activating the Wnt-PCP-JNK signaling pathway and promoting NSCLC malignancy.^626^ LRP8 also plays a significant role in facilitating NSCLC cell proliferation and invasion through the Wnt/β-catenin pathway.^627^ In SCLC, ASPM enhances stemness and invasiveness by stabilizing the expression of GLI1, Dvl3, and SMO, thereby activating both Hh and Wnt signaling pathways.^628^ In lung cancer cells, HORMAD1 promotes EMT by increasing AKT and GSK3β phosphorylation, leading to reduced phosphorylation at Ser33/37/Thr41 of β-catenin and promoting its accumulation and transcriptional activity.^629^ In pericytes, CD248 derepresses Wnt signaling, upregulating angiogenic factors osteopontin and SERPINE1, which support angiogenesis and tumor growth in lung cancer.^630^ Recently, Li et al. developed a humanized antibody, SHH002-hu1, targeting Fzd7, which inhibits NSCLC invasion and metastasis by disrupting Wnt/β-catenin signaling.^631^ This offers a promising therapeutic strategy for lung cancer treatment.

#### Leukemia

Acute lymphoblastic leukemia (ALL) is a malignant hematologic tumor characterized by the uncontrolled proliferation of immature lymphocytes, which includes both B-cell acute lymphoblastic leukemia (B-ALL) and T-cell acute lymphoblastic leukemia (T-ALL). Approximately 80% of ALL cases are classified as B-ALL, which typically has a more favorable prognosis compared to T-ALL.^632^ Based on a zebrafish model of ALL, the Wnt signaling pathway has been implicated as a potential genetic driver of leukemia stem cell fate.^633^ In BCP-ALL with the TCF3-PBX1 fusion gene, Wnt5a is upregulated and cooperates with ROR1 to synergistically activate RhoA/Rac1 GTPases, promoting the proliferation of TCF3-PBX1-positive B-ALL cells.^634^ A 2023 study revealed that HBO1, a potent regulator of CTNNB1, activates the Wnt/β-catenin signaling pathway in B-ALL, driving cell proliferation.^635^ Additionally, overexpression of β-catenin, Notch1, and Notch2 was observed in patients with T-ALL and strongly correlated with the maintenance of stem cell-like phenotypes.^636^

AML and chronic myeloid leukemia (CML) are common leukemias in adults. In AML, high expression of Wnt2b and Wnt11 is associated with poor prognosis, while Wnt10a is linked to a more favorable outcome.^637^ PRICKLE1, a key component of the non-canonical Wnt/PCP pathway, has been reported to be highly expressed in FLT3/DNMT3A/IDH1/IDH2-mutant AML, which is related to poor outcomes.^638^ T-cell immunoglobulin mucin-3 facilitates the recruitment of hematopoietic cell kinase, which phosphorylates p120-catenin and promotes LRP6 formation, thereby activating β-catenin and sustaining cancer stemness in AML.^639^ Jiang et al. found that targeting both Wnt/β-catenin and FLT3 in FLT3-mutant AML can exert strong anti-leukemic effects.^640^ In CML, Wnt3 transcription is regulated by zinc finger protein X-linked, promoting stem/progenitor cell proliferation and resistance to imatinib mesylate.^641^ Moreover, in imatinib mesylate-resistant K562 cells, Wnt2 signaling mediates protective autophagy, which can be suppressed by miR-199a/b-5p.^642^

#### Breast cancer

Breast cancer (BC) remains one of the most common malignancies among women, with millions of cases diagnosed each year, making it the fifth leading cause of cancer-related deaths.^643^ TNBC, accounting for about 20% of all BC cases, is more prevalent in women under 40 and is characterized by aggressive behavior and poor prognosis. Wnt3a signaling has been identified as a key factor influencing estrogen action in TNBC, with LEF1 and TCF4 also playing significant roles.^644^ Additionally, the Wnt3a/GSK3β/β-catenin pathway has been implicated in memory impairment in patients with BC undergoing doxorubicin chemotherapy.^645^ Wnt1-inducible signaling pathway protein-1 is highly expressed in BC tissues, promoting tumor growth and EMT.^646^ Investigating interconal communication in TNBC, Li et al. found that exosomes from low-metastatic subclones facilitate lung metastasis in highly metastatic subclones, with Wnt7a being a critical mediator in this process.^647^ Additionally, DEP domain-containing protein 1B (DEPDC1B) enhances BC cell invasion and migration by mediating β-catenin deubiquitination.^648^ Chemokine-like factor MARVEL transmembrane domain-containing 7 (CMTM7) inhibits BC progression through the miR-182-5p/CMTM7/CTNNA1/β-catenin/TCF3 feedback loop.^649^ PLA2G7 upregulation, a potential negative regulator of the Wnt pathway, may exert protective effects in BRCA1-mutated BC.^650^ Interestingly, a recent study suggested that RSPO3, despite being part of the Wnt pathway, may exert its carcinogenic effects in BC independent of Wnt signaling.^651^

#### Melanoma

Melanoma, a highly malignant tumor originating from melanocytes, is closely linked to the activation of the Wnt signaling pathway, which plays a significant role in metastasis and invasion. Increased Wnt signaling in normal skin cells or less aggressive melanoma cells can induce EMT, leading to a more aggressive phenotype.^652^ In the melanoma microenvironment, myeloid-derived suppressor cells are the primary source of Wnt5a, which contributes to immunosuppression and metastasis promotion.^653^ In patients with melanoma carrying BRAF mutations, poorer prognosis is associated with the Wnt5a-ROR2 axis-mediated secretion of vascular endothelial growth factor and altered vascular distribution.^654^ RNF43, a negative regulator of Wnt/β-catenin signaling, also inhibits non-canonical Wnt5a signaling, thereby suppressing melanoma invasion and enhancing response to targeted therapies.^655^ Fzd6-mediated Wnt/β-catenin signaling significantly increases melanoma cell invasiveness *via* EMT but does not promote cell proliferation.^656^ Targeting Wnt/β-catenin signaling can also exacerbate ferroptosis by modulating microphthalmia-associated transcription factor, improving the efficacy of anti-PD-1 immunotherapy in melanoma.^657^ Furthermore, knocking out RIPK4 in melanoma cells blocks Wnt3a-induced LRP6 and β-catenin activation, inhibiting melanoma growth, migration, and invasion.^658^ Overall, the Wnt signaling pathway, especially Wnt5a, plays a pivotal role in melanoma biology and offers promising targets for therapeutic interventions.

#### Glioblastoma multiforme

Glioblastoma multiforme (GBM) is a highly aggressive malignant tumor of the central nervous system with a complex pathogenesis and poor prognosis. The Wnt signaling pathway plays a critical role in the renewal and differentiation of GBM stem cells (GSCs), significantly impacting tumor progression.^659^ In human GSCs, overexpression of Wnt5a has been linked to the promotion of tumor-promoting stem-like characteristics and is a key regulator of brain invasion.^660^ Wnt6 has been identified as an oncogene in GBM and serves as an independent prognostic biomarker for patient survival.^661^ Norrin, a Wnt ligand, binds to Fzd4, activating the canonical Wnt pathway and inhibiting the growth of GSCs with low ASCL1 expression.^662^ Components of the non-canonical Wnt/PCP signaling pathway, including Vangl1 and Fzd7, are involved in Rho GTPases-mediated actin cytoskeletal rearrangement, promoting GBM cell proliferation, migration, and invasion.^663^ Re-expression of the WIF1 has been shown to suppress Wnt/Ca^2+^ pathway activation mediated by Wnt5a, by downregulating lncRNA MALAT1, which inhibits GBM cell migration.^664^ A recent study highlighted that PRMT6 promotes EMT in GBM through the activation of the Wnt/β-catenin pathway *via* YTHDF2.^665^ Targeting the Wnt/β-catenin pathway also reduces the secretion of neuroligin 3 into the TME, thereby inhibiting CSLC properties in GBM.^666^ Additionally, the Wnt pathway contributes to chemoresistance in GBM through complex mechanisms involving autophagy or endothelial transformation into mesenchymal stem-like cells.^667,668^ A 2023 study revealed that EZH2 and HP1BP3 epigenetically activate Wnt7b, increasing resistance to temozolomide in GBM cells.^669^

**Fig. 7:**
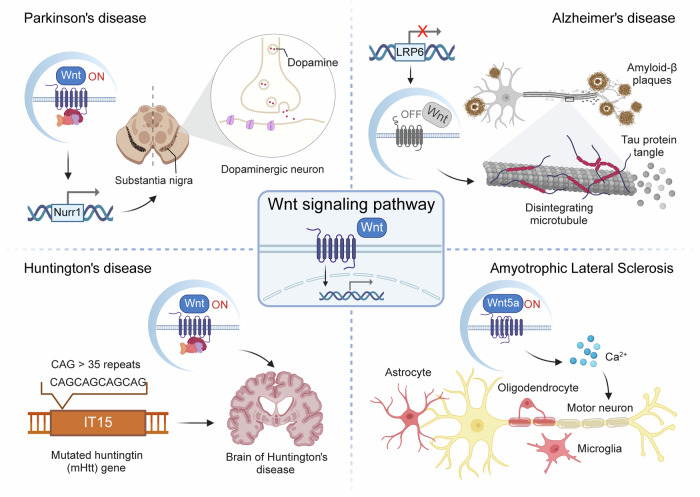
Dysregulated Wnt signaling is linked to several human neurodegenerative diseases, including Parkinson’s disease (PD), Alzheimer’s disease (AD), Huntington’s disease (HD), and amyotrophic lateral sclerosis (ALS). In PD, Wnt signaling could active nuclear receptor-related 1 (Nurr1), a transcription factor critical for the development, differentiation, and maintenance of midbrain dopaminergic neurons. In AD, the deletion of the neuronal LRP6 gene results in the downregulation of Wnt signaling, leading to amyloid pathology. In HD, Wnt/β-catenin signaling is activated in human HD brain tissue. Additionally, Wnt5a has been shown to protect motor neurons through the non-canonical Wnt/Ca2^+^ signaling pathway in ALS. Image created with BioRender (https://biorender.com/)

**Fig. 8:**
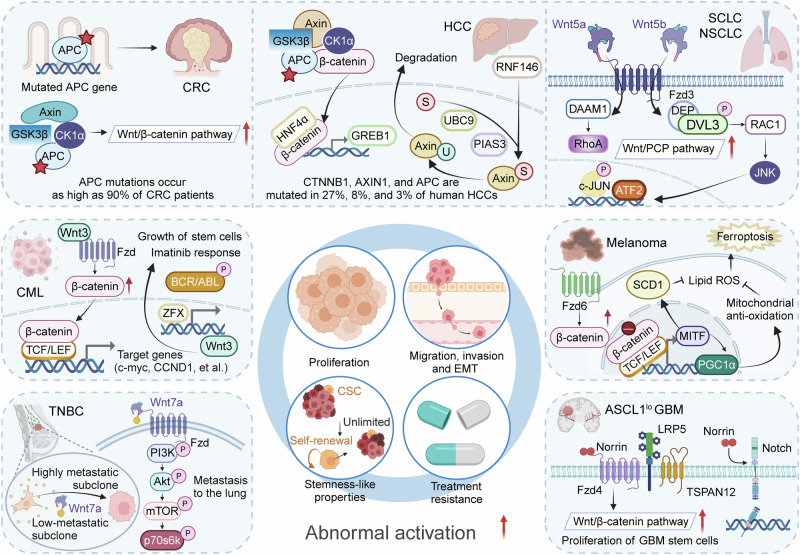
Overview of the Wnt signaling pathway in various cancers, including colorectal cancer, hepatocellular carcinoma, lung cancers, chronic myeloid leukemia, triple-negative breast cancer, melanoma, and glioblastoma multiforme. Hyperactivation of Wnt signal, as a driving factor of cancers, affects tumor proliferation, invasion, migration, EMT, Stemness-like properties and drug resistance. CK1α casein kinase 1α, APC adenomatous polyposis coli, GSK3β glycogen synthase kinase 3β, HNF4α hepatocyte nuclear factor α, GREB1 growth regulation by estrogen in breast cancer 1, RNF146 RING finger protein 146, UBC9 ubiquitin-conjugating enzyme 9, PIAS3 protein inhibitor of activated STAT3, DAAM1 disheveled-associated activator of morphogenesis 1, JNK c-Jun N-terminal kinase, DVL3 disheveled 3, ZFX zinc finger protein X-linked, BCR/ABL breakpoint cluster region-c-Abelson murine leukemia viral oncogene homolog, MITF microphthalmia-associated transcription factor, PGC1α peroxisome proliferator-activated receptor gamma coactivator 1α, PI3K phosphatidylinositol 3-kinase, mTOR mammalian target of rapamycin, EMT epithelial-mesenchymal transition, PCP planar cell polarity, LRP5 lipoprotein receptor-related protein 5, TCF/LEF T cell factor/lymphoid enhancer factor, TSPAN12 tetraspanin 12, ASCL1 achaete-scute family bHLH transcription factor 1, APC Adenomatous polyposis coli, CRC Colorectal cancer, HCC Hepatocellular carcinoma, SCLC Small cell lung cancer, NSCLC Non-small cell lung cancer, TNBC Triple-negative breast cancer, CML Chronic myeloid leukemia, GBM Glioblastoma multiforme. Image created with BioRender (https://biorender.com/)

In summary, this review comprehensively outlines the regulatory mechanisms and pathological roles of the Wnt signaling pathway in a variety of disease contexts (Fig. 7, Fig. 8 and Table 3).

**Table 3:** Mechanisms of Wnt signaling pathway in various diseases

Disease	Wnt signaling component	Function	Mechanism	Related molecule	Cell	Refs.
Atherosclerosis	Wnt4	Accelerate endothelial cell dysfunction	circUSP36→adsorbe miR-637→Wnt4↑	circUSP36, miR-637	Human aortic endothelial cells	^409^
	Wnt5a	Protect against atherosclerosis	Wnt5a → PI3K/Akt/mTORC1 ↓ →lysosomal cholesterol egress↑	PI3K, Akt, mTORC1, NPC1, NPC2	Human vascular smooth muscle cells, human embryonic kidney cells	^415^
	Wnt5a	Regulate cholesterol homeostasis and inflammatory response	Wnt5a/Ror2/ABCA1 signaling pathway	Ror2, ABCA1	Vascular smooth muscle cells	^416^
	β-catenin	Promote vascular remodeling	β-catenin C-terminal→S1PR1 transcription ↑ →SMCs proliferation↑	S1PR1	Primary mouse aortic SMCs	^418^
	Wnt3a	Atherosclerosis resolution	Wnt3a → PGE2/STAT3 ↑ →macrophage responses to IL-4↑	STAT3, IL-4, PGE2	Bone marrow-derived macrophages	^421^
Atrial fibrillation	Wnt5a	Promote myocardial fibrosis	LncRNA HOTAIR→recruit PTBP1→stability of Wnt5a↑	LncRNA HOTAIR, PTBP1	Primary atrial fibroblasts	^429^
Arrhythmogenic cardiomyopathy	GSK3β, Wnt, β-catenin	Regulate cardiac sodium channel	GSK3β ↓ →Wnt/β-catenin ↑ →sodium current density↑	PKP2	iPSC, HEK293T cell line	^436^
Arrhythmogenic right ventricular cardiomyopathy	Wnt, β-catenin, TCF, LEF1	Recapitulate the phenotype of ARVC	Nuclear plakoglobin→Wnt/β-catenin↓	Nuclear plakoglobin	HL-1 cardiac myocytes	^441^
Myocardial infarction	Wnt2, Wnt4, β-catenin, Fzd4/2, LRP6	Promote cardiac fibrosis	Wnt2 and Wnt4 ↑ →Fzd4/2 and LRP6 ↑ →β-catenin/NF-κB ↑	NF-κB	Primary neonatal rat cardiomyocytes and cardiac fibroblasts	^260^
	Wnt3a	Protect myocardial Injury	Wnt3a→Cys-C/ROS signaling ↓ →mitochondrial damage↓	Cys-C, ROS	Cardiomyocytes	^451^
	Wnt5a-PCP	Improve wound repair and prevent cardiac rupture	TGFβ1-Smad2/3 signaling axis ↑ →CTHRC1 ↑ →Wnt5a-PCP signaling↑	Cthrc1, TGFβ1, smad2/3	Primary cardiac fibroblasts	^452^
	Wnt, β-catenin	Inhibit EndMT	sFRP3 ↑ →Wnt/β-catenin ↓ →FOXM1 ↓ →EndMT↓	sFRP3, FOXM1	Ovine mitral VEC, ovine carotid artery endothelial cells	^457^
	Wnt5a	Alleviate myocardial injury	sFRP5 ↑ →Wnt5a/JNK ↓ →angiogenesis of HUVECs↑	sFRP5, p-JNK1/2/3	HUVECs	^458^
Parkinson’s disease	Wnt, β-catenin, GSK-3β	Neuroprotection and repair	GSK-3β ↓ →Wnt/β-catenin ↑ →nerve repair↑	GSK-3β	Primary neural stem/precursor cell	^476^
	Wnt, β-catenin	Impact midbrain dopaminergic neuron specification	Wnt/β-catenin ↓ →NR0B1 ↑ → RSPO2↓	NR0B1, RSPO2	hESC line	^477^
	Wnt, β-catenin	Improve hippocampal neurogenesis	Dopamine D1 receptor ↑ →Wnt/β-catenin↑	Dopamine D1 receptor	/	^478^
	Wnt, β-catenin	Promote mitochondrial biogenesis and dopaminergic neurogenesis	Axin-2 ↓ →Wnt/β-catenin↑	Axin-2	/	^479^
Alzheimer’s disease	LRP6, Wnt	Synaptic abnormalities and amyloid pathology	LRP6 ↓ →Wnt signaling↓	Amyloid-β	SH-SY5Y cells, SH-SY5Y-APP, HEK293 cells, N2a cells	^489^
	Wnt, β-catenin	Mitigate pathogenic phenotypes of Alzheimer’s disease	CXXC5 ↓ →Wnt/β-catenin↑	CXXC5	/	^500^
	Wnt, β-catenin	Negate the neuronal dysregulation	Xmu-mp-1 ↑ → MST/Hippo signaling ↓ →Wnt/β-catenin↑	Xmu-mp-1, MST, Hippo	/	^501^
	Wnt, GSK-3β	Restore synapse integrity and memory	Dickkopf-3 ↓ →Wnt/GSK-3β ↑ →Wnt/JNK↓	Dickkopf-3, JNK	Primary hippocampal neurons	^502^
	Wnt3a, β-catenin	Mitigate cognitive decline and tauopathy	APOE3Ch ↑ →Wnt3a ↑ →β-catenin↑	APOE3	APOE3Ch cerebral organoids	^503^
Amyotrophic lateral sclerosis	Wnt5a, Ca2^+^	Protect motor neurons	Wnt5a ↑ →Wnt/Ca2^+^↑	SOD1	NSC-34 cell	^522^
Type 2 diabetes mellitus	Wnt, β-catenin, GSK-3β	Promote osteogenesis of bone marrow stromal cells	BMP2 ↑ →Wnt/β-catenin ↑ →GSK-3β ↓	BMP2	Primary bone marrow stromal cells	^537^
	Wnt3a, β-catenin, p-GSK-3β	Neuroprotective and regulate insulin resistance	LVRL ↑ →Wnt3a/β-catenin/p-GSK-3β ↑	LVRL	PC12 cell	^538^
	Wnt, β-catenin	Alleviate cognitive dysfunction	Ex-4 ↑ →Wnt/β-catenin/NeuroD1 ↑ →Ins2-derived brain insulin↑	Ex-4, NeuroD1	HT22 cells, HEK-293T cells	^539^
	β-catenin	Protect against T2DM-induced renal apoptosis	Zn ↑ →Nrf2 ↑ →β-catenin↓	Zn, Nrf2	HK11 cells	^540^
Obesity	Wnt5a, Fzd2, Fzd5	Regulate vascular redox signaling	Wnt5a ↑ →Fzd2/USP17/RAC1 ↑ → NADPH oxidases↑	USP17, RAC1	Primary vascular smooth muscle cell	^546^
Non-alcoholic fatty liver disease	LRP6, Wnt, β-catenin	Affect individual susceptibility to NAFLD	LRP6 ↑ →Wnt/β-catenin-Cyp2e1↑	Cyp2e1	HL7702 cell	^557^
	LRP6, Wnt, β-catenin	Alleviate NAFLD	miR-21 ↓ → LRP6 ↑ →Wnt/β-catenin↑	miR-21	/	^558^
	Wnt, β-catenin	Promote liver regeneration	Sirt1 ↑ →Wnt3a/β-catenin↑	Sirt1	Hepa1-6 cells, WB-F344 stem cells	^561^
Inflammatory bowel disease	Wnt2b	Promote colon inflammatory injury	Wnt2b ↑ →competitively bind to IKIP → NF-κB pathway↑	IKIP, NF-κB	THP1 cells, HEK293T cells	^569^
Crohn’s disease	Wnt2b, Fzd4	Promote intestinal penetrating	Wnt2b ↑ →Fzd4 ↑ →EMT↑	E-cadherin, vimentin	HT29 cells	^572^
Systemic lupus erythematosus	Wnt5a	Promote proliferation and adhesion of CD4^+^ T cells	LINC00176 ↑ → WIF1 ↓ →Wnt5a↑	LINC00176, WIF1	CD4^+^ T cells	^578^
	Wnt, β-catenin	Promote cell senescence in BM-MSCs	Wnt/β-catenin ↑ →p53/p21↑	p53, p21	BM-MSCs	^579^
Lupus nephritis	Wnt, β-catenin	Promote EMT and TIL	CX3CL1 ↑ →Wnt/β-catenin↑	CX3CL1	HK-2 Cell	^582^
Rheumatoid arthritis	Wnt, β-catenin	Promote osteoblasts differentiation	IL-35 ↑ →Wnt/β-catenin↑	IL-35	MC3T3E1 cells	^587^
	Wnt, β-catenin	Promote rheumatoid arthritis synovial fibroblasts proliferation	ASIC1a ↑ →Wnt/β-catenin/c-Myc↑	ASIC1a, c-Myc	Rheumatoid arthritis synovial fibroblasts	^590^
	Wnt, β-catenin	Facilitate FLS aggressive phenotype and disrupted chondrocyte homeostasis	Rspo2 ↑ →Wnt/β-catenin↑	Rspo2	FLS	^591^
Multiple sclerosis	Wnt, β-catenin	Inhibit differentiation of oligodendrocyte progenitor cells	C1q ↑ →Wnt/β-catenin↑	C1q	Oligodendrocyte progenitor cells	^598^
Colorectal cancer	Dvl3	Promote EMT and CSLCs properties	Wnt/β-catenin/c-Myc/SOX2 axis	c-Myc, SOX2	HCT-8, SW620	^607^
	β-catenin	Chemoresistance	Wnt/β‑catenin/IMPDH2 positive feedback circuit	IMPDH2, Caspase 7/8/9	SW620, RKO, HCT116 and HCT8, HEK293T	^608^
	GSK3β	Oncogene	NLRP12/STK38/GSK3β axis→Wnt/β-catenin↓	NLRP12, STK38	MC38	^610^
	β-catenin	Promote metastasis	RP11-417E7.1/THBS2/β-catenin axis→metastasis and M2 macrophage infiltration↑	lncRNA RP11-417E7.1, HMGA1, THBS2	FHC, HCT116, HCT-8, LoVo, DLD1, SW480, SW620, THP-1, HEK 293	^612^
	Wnt3a, Wnt7b, β-catenin	Liver metastasis and stemness-like properties	m6A modified BACE1-AS/miR-214-3p/TUFT1/Wnt signaling axis	BACE1-AS, TUFT1	CCD841 CoN, SW480, HCT116, SW620, LoVo	^154^
Hepatocellular carcinoma	RNF146	Promote HCC progression	RNF146 SUMOylation at K19/K175→Axin degradation ↑ →β-catenin↑	SUMO3, UBC9, PIAS3, SENP1	HeLa, HEK293T, SK-hep1, HCC-LM3	^619^
	GREB1	Oncogene	Wnt signaling→GREB1 → HNF4α/FOXA2	HNF4α	MCF7, HuH7, HLE, HLF, JHH7, PLC/PRF/5, HepG2, Hep3B	^620^
	LEF1	Facilitate HCC progression	ZMIZ2 → LEF1-mediated Wnt/β-catenin pathway↑	ZMIZ2	LO2, Huh7, Hep3B, HCCLM3, HepG2, SK-hep1, MHCC-97 H	^621^
	Fzd10	Promote expansion of liver CSLCs and lenvatinib resistance	Fzd10-β-catenin/YAP1 axis, Fzd10-β-catenin/c-Jun axis, Fzd10/β-catenin/c-Jun/MEK/ERK axis	METTL3, YAP1	Hep3B, Huh7, SNU398	^622^
Small cell lung cancer	Wnt5a	Oncogene	p130-Wnt5a-RHOA pathway	RHOA	293T, NCI-H69, NCI-H82, NCI-H209, NCI-H524, NCI-H2141, NCI-H2171, NCI-H211, NCI-H526, NCI-H1048, NCI-A549, NCI-H1650, NCI-H2009	^625^
	Dvl3	Drive tumor stemness and progression	Wnt-DVL3-β-catenin signaling axis	ASPM, GLI1, SMO	NCI-H209, NCI-H146, NCI-H1618	^628^
Non-small cell lung cancer	Wnt5b	Oncogene	Fzd3-DVL3-RAC1-PCP-JNK pathway	/	HBE, A549, SPC, H157, H460, LTE	^626^
	LRP8	Proliferation and invasion	LRP8→Wnt/β-catenin signaling	/	95-D, H1299, H460, HCC-827, A549, PC-9, H1975, HBE	^627^
	Fzd7	EMT	SHH002-hu1→Fzd7→Wnt/β-catenin↓	/	BEAS-2B, HEK293T, A549, H1299, H1975	^631^
B-cell acute lymphoblastic leukemia	Wnt5a	Enhance proliferation of TCF3-PBX1 cells	Wnt5a-ROR1→RhoA/Rac1 GTPases ↑ →STAT3↑	ROR1, RhoA	697, RCH-ACV, Kasumi-2 (TCF3-PBX1 positive), REH, Nalm-6, HS-5	^634^
	β-catenin	Oncogene	HBO1 ↑ → H3K14, H4K8, H4K12→Wnt/β-catenin↑	HBO1	NALM-6, REH, RS4;11, Jurkat, KG1a, NB4, MV4–11, THP-1, Raji, Daudi, Jeko-1, NAMALWA	^635^
Acute myeloid leukemia	PRICKLE1	Poor prognosis	PRICKLE1 ↑ →Wnt/PCP	FLT3/DNMT3A/IDH1/IDH2	K562, K562/ADR, THP1, HL60, HL60/ADR, GM12878, MOLM13, MV4-11	^638^
	LRP6	Maintain cancer stemness	TIM-3/HCK/p120-catenin→β-catenin↑	TIM-3, Gal-9	Human cord blood cells, KASUMI-3, Human bone marrow primary cells	^639^
Chronic myeloid leukemia	Wnt3	Modulate the growth and IM response of CML stem/progenitor cells	ZFX ↑ →Wnt3/β-catenin↑	ZFX	K562, 293 T, BaF3	^641^
	Wnt2	Protective autophagy	microRNA-199a/b-5p ↑ →Wnt2↓	microRNA-199a/b-5p	K562, K562R, KU812, KU812R	^642^
Breast cancer	Wnt3a	Doxorubicin-induced memory impairment	Wnt3a/GSK3 β/β-catenin	PI3K, Akt	/	^645^
	β-catenin	Promote metastasis	DEPDC1B → USP5→deubiquitination of β-catenin	DEPDC1B, USP5	MCF-10A, MDA-MB-231, MDA-MB-468, MDA-MB-157, BT-549, HEK-293T, Hs578T	^648^
	β-catenin	Promote progression	A feedback loop of miR-182-5p, CMTM7, CTNNA1, β-catenin, and TCF3	CMTM7	MCF-10A, MDA-MB-231, MCF-7, SK-BR3, T47D, Cal51	^649^
Triple-negative breast cancer	Wnt7a	Promote lung metastasis	Wnt and PI3K/Akt/mTOR	PI3K, Akt, mTOR	4T1	^647^
Melanoma	Wnt5a	Suppress invasion and resistance	RNF43 → VANGL2, ROR1, ROR2→Wnt5a↓	RNF43	T-REx-293, A375, A2058, MelJuSo	^655^
	Fzd6	Promote EMT and invasion	Wnt/β-catenin	/	A375, G361, Hs294T, SK-Mel28, MEL-ST, 451Lu, WM35, WM115	^656^
	β-catenin	Regulate ferroptosis	Wnt/β-catenin ↓ →MITF → PGC1α, SCD1	MITF, PGC1α, SCD1	A2058, A375, B16F10	^657^
Glioblastoma multiforme	Norrin	Inhibit growth in ASCL1^lo^ GSCs	Norrin→Fzd4→Wnt/β-catenin	ASCL1	Primary tumor–derived GSC and hNSC, G523, G472, G440, G411, G564, hNSC-1, hNSC-3	^662^
	Wnt5a	Enhance the migratory potential	WIF1 ↑ →Wnt5a ↓ →Wnt/Ca^2+^ pathway and MALAT1↓	WIF1, MALAT1	LN-229, LN-319, LN-18, LN-428, LN-2669GS	^664^
	β-catenin	Promote migration, invasion, and EMT	PRMT6-YTHDF2-Wnt-β-Catenin axis	PRMT6, CDK9, YTHDF2	LN229, U251MG, U87MG, U118MG, HEB, HEK293T	^665^
	β-catenin	Promote CSLCs properties	Wnt/β-catenin→NLGN3	NLGN3	LN18, LN229, A172, U87MG, U251, U373	^666^

*ABCA1* adenosine triphosphate-binding cassette transporter A1, *Akt* protein kinase B, *ARVC* arrhythmogenic right ventricular cardiomyopathy, *BM-MSCs* bone marrow-derived mesenchymal stem cells, *CML* chronic myeloid leukemia, *CSLC* cancer stem-like cell, *Dvl* disheveled, *EMT* epithelial–mesenchymal transition, *FLS* fibroblast-like synoviocytes, *FOXM1* Forkhead box M1, *Fzd* frizzled, *GSCs* glioblastoma multiforme stem cells, *GSK3β* glycogen synthase kinase 3β, *HCC* hepatocellular carcinoma, *HUVECs* human umbilical vein endothelial cells, *IKIP* IκB kinase-interacting protein, *iPSC* induced pluripotent stem cells, *JNK* Jun N-terminal kinase, *LRP* lipoprotein receptor-related protein, *LVRL* Leu-Val-Arg-Leu, *mTORC1* mechanistic target of rapamycin complex 1, *NAFLD* non-alcoholic fatty liver disease, *NF-κB* nuclear factor-kappa B, *NPC1* Niemann-Pick C1, *NPC2* Niemann-Pick C2, *PCP* planar cell polarity, *PGE2* prostaglandin E2, *PI3K* phosphatidylinositol 3-kinase, *PKP2* plakophilin-2, *PTBP1* polypyrimidine tract-binding protein 1, *ROR2* receptor tyrosine kinase-like orphan receptor 2, *S1PR1* sphingosine-1-phosphate receptor 1, *sFRP* secreted Frizzled-related protein, *SMCs* smooth muscle cells, *STAT* signal transducer and activator of transcription, *T2DM* type 2 diabetes mellitus, *TGF-β* transforming growth factor-β, *TIL* tubulointerstitial lesions, *ZFX* zinc finger protein X-linked

## Therapeutic targeting of wnt signaling

Although the Wnt signaling pathway is essential for maintaining normal biological functions, its dysregulation can precipitate the onset of various diseases, which poses a considerable threat to public health and social stability. As a result, the scientific community has increasingly focused on identifying key targets within the Wnt signaling pathway, with the goal of developing novel therapeutic strategies, particularly for cancer treatment. Beyond oncology, inhibiting Wnt signaling has demonstrated therapeutic potential in non-cancerous conditions. For example, sclerostin, a negative regulator of Wnt signaling expressed specifically in bone tissue, has been extensively studied in the context of osteoporosis. Both zoledronic acid and denosumab reduce bone resorption by upregulating endogenous Wnt inhibitors such as SOST and DKK1.^670,671^ Additionally, romosozumab, a monoclonal antibody targeting SOST, has yielded promising results in clinical trials (NCT01575834).^672^ Moreover, activating the Wnt pathway to promote tissue regeneration presents a compelling clinical opportunity. Certain small molecule compounds, including L807mts, SB-216763, Bio, and CHIR, have been shown to enhance the transcription and expression of Wnt target genes by inhibiting GSK3β, which may offer therapeutic benefits for neurodegenerative diseases such as Alzheimer’s disease.^673^

**Fig. 9:**
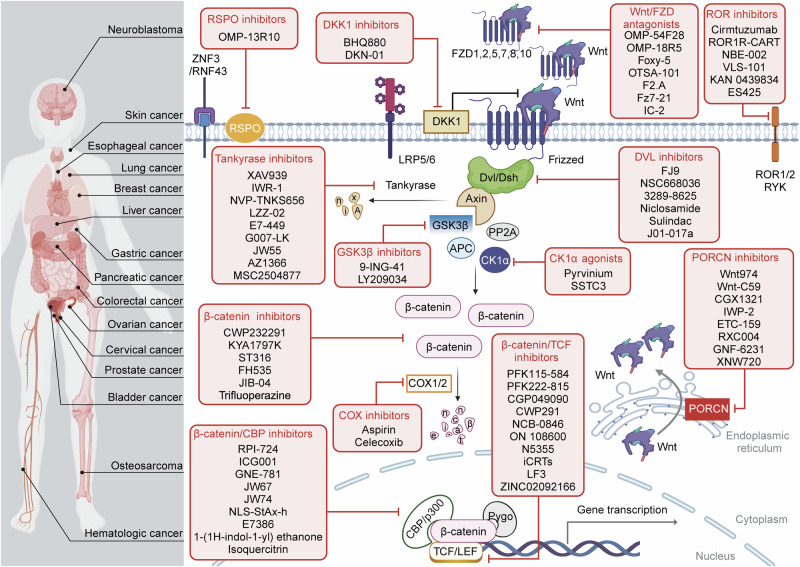
Small molecule therapeutic drug map targeting all parts of the Wnt pathway. On the left side of the picture are the tumor types that can be treated with small molecule drugs, and on the right side of the picture are the various categories of small molecule drugs. RSPO R-spondin, DKK Dickkopf, FZD Frizzled, ROR receptor tyrosine kinase-like orphan receptor, LRP lipoprotein receptor-related protein, Dvl/Dsh disheveled, GSK3β glycogen synthase kinase 3β, APC adenomatous polyposis coli, PP2A protein phosphatase 2A, CK1α casein kinase 1α, ROR1/2 receptor tyrosine kinase-like orphan receptor 1 and 2, RYK tyrosine kinases, PORCN Porcupine, COX cyclooxygenase, CBP cyclic AMP response element-binding protein, TCF/LEF T cell factor/lymphoid enhancer factor. Image created with BioRender (https://biorender.com/)

As previously outlined, the Wnt signaling pathway is a complex network of signal transduction pathways initiated by the binding of Wnt ligands to membrane receptors, which subsequently activate multiple downstream channels. This pathway involves a wide array of components, and interventions at any point within the pathway can influence the entire signaling cascade. This review comprehensively summarizes the most recent advancements in disease treatment by targeting key components of the Wnt signaling pathway, including Wnt ligands/receptors, the β-catenin destruction complex, and the β-catenin/TCF transcription complex. Notably, this review emphasizes the development of small-molecule inhibitors, monoclonal antibodies, and combination therapy strategies (Fig. 9, Table 4 and Table 5).

**Table 4:** Small molecule inhibitors and monoclonal antibodies targeting the Wnt pathway

Name	Targets	Functional effects	Cancer type	Clinical Trials	Refs.
Wnt-C59	PORCN	Inhibited stemness properties of NPC cells in a dosage-dependent manner	NPC	/	^674^
LGK974 (Wnt974)	PORCN	/	HNSCC	Phase II NCT02649530	/
		Inhibited Wnt secretion and signaling and enhanced targeting of CML stem cells while sparing their normal counterparts	CML	/	^675^
		Inhibited tumor growth, prevented ascites formation, and prolonged survival in mouse models	EOC	/	^676^
ETC-159	PORCN	Inhibition of PORCN in RSPO3-translocated cancers caused a marked remodeling of the transcriptome, with loss of cell cycle, stem cell and proliferation genes, and an increase in differentiation markers	CRC	/	^677^
GNF-1331/GNF-6231	PORCN	Demonstrated potent inhibition activities and induced robust anti-tumor efficacy in a BC mouse model	BC	/	^678^
CGX1321	PORCN	/	Solid tumors	Phase I NCT03507998	/
		Manipulating the Wnt/β-catenin signaling pathway to promote anti-tumor immune infiltration into the TME to sensitize ovarian cancer to ICB therapy CGX1321(Wnt inhibitor) increased infiltrating CD8^+^ T cells in the TME and decreased tumour burden	OC	/	^679^
3289–8625	PDZ domain of DVL	Suppressed the growth of PCa cells	PCa	/	^682^
OMP-18R5 (Vanticumab)	Fzd1, Fzd2, Fzd5, Fzd7 and Fzd8	/	Solid tumors	Phase I NCT01345201	/
		Reduced growth of HNSCC patient-derived xenografts and suppressed Wnt activation at the tumor epithelial-stromal boundary	HNSCC	/	^688^
OMP-54F28 (Ipafricept)	Fzd8	/	Advanced solid tumours	Phase I NCT01608867	/
		Reduced growth of HNSCC patient-derived xenografts and suppressed Wnt activation at the tumor epithelial-stromal boundary	HNSCC	/	^688^
Foxy-5	Fzd5	/	BC, CRC, PCa	Phase I NCT02020291	/
OTSA-101	Fzd10	/	SS	Phase I NCT01469975	/
JW67/JW74	β-catenin AXIN2, SP5 and NKD1	Suppressed in vitro proliferation of CRC cell	CRC	/	^693^
JW74	Tankyrase AXIN2	Reduced cell growth and differentiation of OS cells	OS	/	^694^
JW55	Tankyrase	Decreased canonical Wnt signaling in CRC cells and reduced tumor growth	CRC	/	^695^
LZZ-02	Tankyrase	Inhibited the growth of CRC cell harboring constitutively active β-catenin	CRC	/	^696^
XAV939	Tankyrase	Stimulating beta-catenin degradation by stabilizing AXIN via inhibiting the poly-ADP-ribosylating enzymes tankyrase 1 and tankyrase 2	/	/	^697^
	Tankyrase	Blocked Wnt/β-catenin signaling and reduced the expression of anti-apoptosis protein	NB	/	^698^
	Tankyrase	Enhanced radiosensitivity	CC	/	^699^
	Tankyrase 1	Increased chemosensitivity in colon cancer cell lines	CRC	/	^700^
IWR-1	Tankyrase	Inhibited the growth of a subcutaneous human osteosarcoma xenograft in vivo	Osteosarcoma	/	^701^
Pyrvinium	CK1α	Attenuated the levels of Wnt-driven biomarkers and inhibited adenoma formation in APC^min^ mice	CRC	/	^703–705^
		Enhanced sensitivity of OC cells to chemotherapy	OC	/	^706^
		Inhibited the self-renewal and metastasis of BC stem cells	BC	/	^707^
SSTC3	CK1α	Inhibited the growth of CRC xenografts in mice	CRC	/	^708^
		Attenuated growth and metastasis of orthotopic patient-derived TRP53-mutant, MYCN-amplified, SHH subgroup medulloblastoma xenografts, increasing overall survival	Medulloblastoma	/	^709^
ICG-001	CBP/β-Catenin	Binding CBP and disrupting its interaction with β-catenin, and is effective in killing tumour cells both in vitro experiments and mouse xenograft models of CRC and PDAC	CRC, PDAC	/	^710,711^
PRI-724	CBP/β-Catenin	/	Advanced solid tumors	Phase I NCT01302405	/
		/	PC	Phase I NCT01764477	/
		/	AML, CML	Phase II NCT01606579	/
		An active enantiomer of ICG-001, has already entered Phase I clinical trials for treating CRC and PDAC	CRC, PDAC	/	^712^
		Increased sensitization to platinum chemotherapy	EOC	/	^713^
E7386	CBP/β-Catenin	The first-in-class orally active β-catenin-CBP antagonist that inhibited Wnt/β-catenin pathway in PDX model of HCC	HCC	Phase I NCT03833700 NCT03833700	^714^
GNE-781	CBP/β-Catenin	Displayed antitumor activity in an AML tumor model and decreased Foxp3 transcript levels in a dose dependent manner	AML	/	^715^
1-(1H-indol-1-yl) ethanone	CBP/EP300	Inhibited cell growth in several PCa cell lines	PCa	/	^716^
Isoquercitrin	CBP downstream of β-catenin translocation to the nuclei	Inhibited tumor cells, but had no effect on normal cells	CRC	/	^717^
CPG049090, PKF115-584 and PKF222-815	β-catenin/TCF complex	Disrupted the interaction of β-catenin/TCF complex, suppressing the proliferation of CRC cells in vitro assays	CRC	/	^718^
PKF115-584	β-catenin/TCF complex	Restored immunocompetence which suppressed by β-catenin activation	Melanoma	/	^378^
NCB-0846 (TNIK inhibitor)	β-catenin and TCF4 transcription complex	Reduced tumour formation	CRC	/	^719,720^
ON 108600 (CK2/TNIK dual inhibitor)	β-catenin and TCF4 transcription complex	Exhibited significant killing activity against paclitaxel-resistant TNBC cell lines displaying a stem-like phenotype	TNBC	/	/
N5355 (Aminothiazole-based TNIK inhibitor)	β-catenin and TCF4 transcription complex	Killed only Wnt-dependent cancer cells but had no effect on the viability of Wnt-independent cell lines	CRC	/	/
iCRTs	β-catenin/TCF4 transcription complex	Enhanced the infiltration of T and NK cells in syngeneic mouse models of CRC by blocking β-catenin/TCF interaction	HNCSC, hypopharynx cancer, CRC	/	^385,723,724^
CCT036477, iCRT14, or PKF118-310	β-catenin/TCF4 transcription complex	/	MCL	/	^725^
iCRT14	β-catenin/TCF4 transcription complex	Improved chemosensitivity	ALL	/	^726^
iCRT3	β-catenin/TCF4 transcription complex	Increased apoptosis in vitro	TNBC	/	^727^
iCRT14	β-catenin/TCF4 transcription complex	Suppressed CCL28 expression and Treg cell infiltration in the stomach	GC	/	^728^
LF3	β-catenin and TCF4 transcription complex	Suppressed features of cancer cells related to Wnt signaling, including high cell motility, cell-cycle progression, and the overexpression of Wnt target genes	CRC	/	^721^
ZINC02092166	β-catenin/TCF complex	Downregulated the expression of Wnt target genes and inhibited the growth of CRC cells	CRC	/	^729^
Trifluoperazine	Wnt/β-catenin	Inhibited cancer stem cell growth and overcame drug resistance of lung cancer	Lung cancer	/	^738^
IC-2	Wnt	Suppressed proliferation and induced apoptosis of bladder cancer cells	Bladder cancer	/	^739^
JIB-04	Wnt/β-catenin	Attenuated CSC tumorsphere formation, growth/relapse, invasion, and migration in vitro	CRC	/	^740^
FH535	Wnt/β-catenin	Inhibited proliferation	HCC	/	^741^
CWP232291	β-catenin SAM68		AML MDS	Phase I NCT01398462	^730^
KYA1797K	β-catenin and Ras	Suppressed the growth of CRCs harboring APC and KRAS mutations, as shown by various in vitro studies and by in vivo studies using xenograft and transgenic mouse models of tumors induced by APC and KRAS mutations	CRC	/	^734,735^
		Inhibited the proliferation and the metastatic capability of stable cell lines as well as patient-derived cells established from TNBC patient tissues	TNBC	/	^736^
M-110 OICR623 (acyl hydrazones)	AXIN2 and SP5	Inhibited constitutive Wnt signaling and blocked the growth of CRC cell lines	CRC	/	^737^
Decitabine	/	Induced DNA demethylation of Wnt/β-catenin pathway, restored the sensitivity of OC patients to carboplatin	OC	Phase II	^731^
Niclosamide	Wnt/β-catenin	/	CRC	phase II NCT02519582	/
	/	/	CRC	phase I NCT02687009	/
	/	Induced apoptosis, impaired metastasis and reduced immunosuppressive cells in BC model	BC	/	^732^
	LRP6	Suppressed cancer cell growth	PCa, BC	/	^733^
ST316	β-catenin and its co-activator, BCL9	/	Advanced solid tumor	phase I/II NCT05848739	/

*ALL* acute lymphoblastic leukemia, *AML* acute myeloid leukemia, *BC* breast cancer, *CC* bervical cancer, *CK1α* casein kinase 1α, *CML* chronic myeloid leukemia, *CRC* colorectal cancer, *Dvl/Dsh* disheveled, *EOC* epithelial ovarian cancer, *GC* gastric cancer, *HCC* hepatocellular carcinoma, *HNSCC* head and neck squamous cell carcinoma, *ICB* immune checkpoint blockade, *MCL* mantle cell lymphoma, *MDS* myelodysplastic syndrome, *NB* neuroblastoma, *NPC* nasopharyngeal carcinoma, *OC* ovarian cancer, *OS* osteosarcoma, *PC* pancreatic cancer, *PCa* prostate cancer, *PDAC* pancreatic ductal adenocarcinoma, *PORCN* porcupine, *SS* synovial sarcomas, *TCF/LEF* T cell factor/lymphoid enhancer factor, *TME* tumor microenvironment, *TNBC* triple-negative breast cancer

**Table 5:** Combination therapies in clinical trials

Name	Combined drugs	Target	Cancer type	Phase	Identifier
WNT974	PDR001	PORCN	PC, CRC, Melanoma, BC, HNSCC, CSCC, ESCC, LSCC	Phase I	NCT01351103
WNT974	LGX818 and Cetuximab	PORCN	CRC	Phase I	NCT02278133
CGX1321	Pembrolizumab	PORCN	Solid tumors, Gastrointestinal cancer	Phase I	NCT02675946
ETC-159	Pembrolizumab	PORCN	Solid tumor	Phase I	NCT02521844
OMP-18R5	Paclitaxel	Fzd receptors	BC	Phase I	NCT01973309
OMP-18R5	Docetaxel	Fzd receptors	Solid tumors	Phase I	NCT01957007
OMP-18R5	Nab-paclitaxel and gemcitabine	Fzd receptors	PC	Phase I	NCT02005315
OMP-54F28	Sorafenib	Fzd8	HCC	Phase I	NCT02069145
OMP-54F28	Paclitaxel and carboplatin	Fzd8	OC	Phase I	NCT02092363
OMP-54F28	Nab-paclitaxel and gemcitabine	Fzd8	PC	Phase I	NCT02050178
PRI-724	Leucovorin calcium, oxaliplatin, or fluorouracil	CBP/β-catenin	CRC	Phase II	NCT02413853
Niclosamide	Enzalutamide	Wnt/β-catenin	PCa	Phase I	NCT02532114
OMP-131R10	FOLFIRI	RSPO3	CRC	Phase I	NCT02482441

*BC* breast cancer, *CBP* cyclic AMP response element-binding protein, *CRC* colorectal cancer, *CSCC* cervical squamous cell cancer, *ESCC* esophageal squamous cell cancer, *HCC* hepatocellular carcinoma, *HNSCC* head and neck squamous cell carcinoma, *LSCC* lung squamous cell cancer, *OC* ovarian cancer, *PC* pancreatic cancer, *PORCN* porcupine, *PCa* prostate cancer

### Targeting Wnt ligand/receptor

#### Porcupine inhibitors

Porcupine (PORCN) is a membrane-bound O-acyltransferase located in the endoplasmic reticulum, responsible for mediating Wnt palmitoylation, a key step for the secretion of Wnt ligands.^90^ Consequently, targeting PORCN to inhibit the production of all active Wnts is an effective strategy to obstruct both autocrine and paracrine Wnt signaling. Extensive research has shown that small molecule inhibitors targeting PORCN can effectively block the Wnt signaling pathway, thereby exerting anti-tumor effects.^674–678^ For example, WNT974 (LGK974) has been demonstrated to reduce the viability of epithelial ovarian cancer (EOC) cells in vitro and to inhibit tumor growth in vivo by blocking Wnt signaling.^676^ Head and neck squamous cell carcinoma (HNSCC) cell lines harboring inactivating Notch1 mutations exhibit sensitivity to LGK974 inhibition. Similarly, ETC-159, another small molecule PORCN inhibitor, has shown efficacy in preclinical models of RSPO-fusion-positive metastatic CRC. In cancers with RSPO3 translocations, PORCN inhibition significantly remodels the transcriptome, leading to a reduction in the expression of genes associated with the cell cycle, stem cell maintenance, and proliferation, while simultaneously increasing the expression of differentiation markers.^677^ Additionally, Wall et al. discovered that CGX-1321, another PORCN inhibitor, enhances anti-tumor immune infiltration within the TME by modulating the Wnt pathway, thereby sensitizing OC to immune checkpoint blockade therapy.^679^ Phase I clinical trials of CGX-1321 have also been initiated, further exploring its potential as a therapeutic agent in cancer treatment.

#### Disheveled inhibitors

Disheveled (Dsh/Dvl) interacts with the carboxyl terminus of the Fzd receptor *via* its PDZ domain, enabling the transmission of Wnt signals to downstream components. Small molecules such as NSC668036, FJ9, and 3289–8625 have been developed to inhibit this interaction, effectively blocking Wnt signal transduction.^680–682^ Among these inhibitors, 3289–8625 has demonstrated significant tumor-suppressive effects, specifically by suppressing the proliferation of prostate cancer cells, highlighting its potential as a therapeutic agent in cancer treatment.^682^

#### Monoclonal antibodies

Targeting the interaction between Wnt ligands and Fzd receptors presents a promising approach for inhibiting the canonical Wnt signaling pathway in cancer therapy. Overexpression of specific Wnt ligands or receptors in various tumors suggests that disrupting these interactions could offer novel therapeutic strategies. Monoclonal antibodies against Wnt-1 and Wnt-2 have shown efficacy in inhibiting the Wnt pathway, leading to tumor suppression in cancers such as melanoma, sarcoma, CRC, and NSCLC.^683,684^ Fzds and LRP5/6 are critical receptors within the Wnt pathway. For instance, selective inhibition of Wnt3 binding to LRP6 *via* a single-chain antibody has been shown to curtail excessive proliferation in intestinal organs of mice with RNF43 and ZNRF3 mutations, while promoting terminal differentiation.^73^ Whole-genome CRISPR screening has identified Fzd5 as an essential Wnt receptor for the survival of PDAC cell lines with RNF43 mutations.^685,686^ Correspondingly, an Fzd5 inhibitory antibody effectively suppressed the growth of RNF43-mutant PDAC cell lines in both in vitro and in vivo models. Similarly, targeting Fzd5 also impaired the survival of CRC organoids with RNF43 mutations, although organoids with APC mutations remained unaffected.

OMP-18R5 (Vanticumab), developed by OncoMed Pharmaceuticals/Bayer, is a monoclonal antibody that targets five Fzd receptors: Fzd1, Fzd2, Fzd5, Fzd7, and Fzd8. Its safety and efficacy are under evaluation in clinical trials for NSCLC, pancreatic cancer, and BC, both as a standalone therapy and in combination with chemotherapy.^687^ OMP-54F28 (Ipafricept, IPA), a recombinant fusion protein consisting of the cysteine-rich domain of Fzd8 linked to a human IgG1 Fc fragment, competes with Fzd8 receptors by binding to Wnt ligands, thus disrupting Wnt signaling. In OC patient-derived xenograft models, OMP-54F28 has shown the ability to reduce CSCs populations, inhibit tumor growth, and promote cellular differentiation. In HNSCC, both OMP-54F28 and OMP-18R5 have demonstrated suppression of patient-derived xenograft growth by inhibiting Wnt activation at the tumor epithelial-stromal interface.^688^ Beyond their direct effects on tumor cells, Wnt pathway inhibitors enhance immune activation and infiltration in the tumor microenvironment, amplifying the overall antitumor response. The Wnt/β-catenin signaling pathway is well-known for its immunosuppressive role in numerous cancers and is now recognized as a valuable target for immunotherapy. In mouse melanoma models, intrinsic β-catenin activity within tumors has been shown to exclude T cells and confer resistance to PD-L1/anti-CTLA-4 monoclonal antibody therapy.^375^ Furthermore, analysis of primary BRAF-mutant melanoma revealed a negative correlation between T cell infiltration and β-catenin levels in tumor cells.^689^ The Wnt3a-β-catenin signaling cascade depletes tumor-infiltrating T cells, reducing their antitumor activity and inhibiting the generation of effector memory T cells.^690^

### Targeting the β‑catenin-destruction complex

#### Tankyrase inhibitors

Tankyrase, a member of the poly (ADP-ribose) polymerase (PARP) family, consists of two isoforms: Tankyrase 1 (PARP5a) and Tankyrase 2 (PARP5b). Both isoforms play a critical role in promoting the degradation of Axin through the ubiquitin-proteasome pathway, which in turn activates the Wnt signaling pathway.^691,692^ The inhibition of Tankyrase has been shown to impede tumor progression, making it a promising target in cancer therapy.^693–701^ XAV939, a well-known Tankyrase inhibitor, functions by inhibiting Tankyrase 1 and 2, thereby stabilizing Axin and promoting the degradation of β-catenin. In neuroblastoma, XAV939 blocks the Wnt/β-catenin signaling pathway and decreases the expression of anti-apoptotic proteins.^698^ In cervical cancer, XAV939 enhances radiosensitivity, while in colon cancer cell lines, it increases chemosensitivity.^699,700^ Structural optimization of XAV939 led to the development of a more selective Tankyrase inhibitor, NVP-TNKS656. Additionally, IWR-1, another Tankyrase inhibitor, has demonstrated the ability to inhibit the growth of human subcutaneous osteosarcoma xenografts in vivo.^701^ However, the clinical application of these inhibitors has been hampered by gastrointestinal toxicity, highlighting the need for further optimization.^702^

#### CK1α activators

CK1α is a critical component in the assembly of the β-catenin destruction complex, and agonists targeting CK1α have shown efficacy in inhibiting Wnt signaling. Research has demonstrated that Pyrvinium, which binds to all members of the CK1 family at low nanomolar concentrations in vitro, selectively enhances CK1α activity. In CRC, Pyrvinium reduces Wnt-driven biomarker levels and inhibits adenoma formation in APC^min^ mice, a model for CRC.^703–705^ Additionally, in OC, Pyrvinium increases the sensitivity of cancer cells to chemotherapy, and in BC, it inhibits the self-renewal and metastasis of CSCs.^706,707^ Another CK1α activator, SSTC3, has also demonstrated tumor-suppressive effects in both CRC and medulloblastoma, suggesting the therapeutic potential of CK1α agonists in various cancers driven by Wnt signaling.^708,709^

### Targeting CBP/β-catenin complex

CBP (Cyclic AMP response element-binding protein) is an intracellular transcription coactivator that plays a pivotal role in regulating transcription. Acting as a coenzyme, CBP interacts with β-catenin to facilitate transcriptional activation. CBP/β-Catenin inhibitors, such as ICG-001, disrupt this interaction between CBP and β-Catenin by binding to CBP, thereby inhibiting β-catenin-mediated transcription. ICG-001 has shown efficacy in targeting tumor cells both in vitro and in vivo, particularly in mouse models of CRC and PDAC.^710,711^ The active enantiomer of ICG-001, PRI-724, undergoes rapid hydrolysis to its active form, C-82, upon phosphorylation in the body. PRI-724 has advanced to Phase I clinical trials for CRC and PDAC, demonstrating an ability to enhance sensitivity to platinum-based chemotherapy in EOC.^712,713^ Other CBP/β-catenin inhibitors currently under investigation include E7386, GNE-781, 1-(1H-indol-1-yl) ethanone, and Isoquercitrin.^714–717^ Notably, Isoquercitrin inhibits the Wnt pathway by interfering with the nuclear transport of β-catenin and has shown selective inhibitory effects on CRC tumor cells without impacting normal cells.^717^

### Targeting β‑catenin/TCF transcription complex

The activation of target gene transcription by the β-catenin/TCF complex represents the final step in the canonical Wnt signaling pathway. Blocking this step by β-catenin/TCF transcription complex inhibitors has the potential to reduce side effects typically associated with upstream intervention. High-throughput ELISA screening has identified eight compounds that interfere with the β-catenin/TCF complex in a dose-dependent manner, including PFK115-584 and CGP049090.^718^ Notably, in melanoma, PFK115-584 also reverses the immunosuppression caused by β-catenin activation.^378^ Other small molecules, such as PNU-74654, NCB-0846, and LF3, competitively bind to β-catenin, preventing its interaction with TCF4.^719–721^ TRAF2 and NCK interacting kinase (TNIK), a key regulator within the TCF4/β-catenin transcriptional complex, is targeted by ON 108600, a CK2/TNIK dual inhibitor that demonstrates significant cytotoxicity against paclitaxel-resistant TNBC cell lines with stem-like characteristics.^722^ N5355, an aminothiazole-based TNIK inhibitor, selectively targets Wnt-dependent cancer cells, sparing Wnt-independent lines.^385,723,724^ Furthermore, β-catenin responsive transcription (CRT) is a key target in oncology, with CRT inhibitors (iCRT3, iCRT5, iCRT14) showing selective cytotoxicity in CRC cells by inhibiting β-catenin-mediated transcription. This interaction between β-catenin and TCF also enhances T and NK cell infiltration, with iCRT compounds proving effective in treating mantle cell lymphoma, ALL, TNBC, and gastric cancer.^721,725–728^ Catrow et al. identified a new small molecule inhibitor, ZINC02092166, which curtails CRC cell growth by downregulating Wnt target genes.^729^

### Others

A variety of other small molecule inhibitors directly target the Wnt/β-catenin pathway, disrupting Wnt signaling and subsequent gene activation. For instance, CWP232291, Decitabine, and Niclosamide are currently in Phase I and II clinical trials.^730–733^ Other inhibitors, including Trifluoperazine, IC-2, JIB-04, FH535, KYA1797K, and M-110 OICR623, have shown efficacy in treating various cancers such as lung cancer, bladder cancer, CRC, HCC, and TNBC.^734–741^

### Combination therapies

Although small molecule inhibitors and monoclonal antibodies have exhibited substantial tumor-suppressive effects, the high doses necessary for effective monotherapy often result in severe adverse effects, including gastrointestinal toxicity, significant weight loss, and increased mortality. However, emerging evidence suggests that lower doses of TNKS inhibitors, when incorporated into combination therapy regimens, can achieve notable anti-tumor efficacy. Thus, the exploration of combination targeted therapies is crucial in identifying optimal therapeutic strategies. Table 5 outlines ongoing clinical trials investigating Wnt pathway inhibitors in combination with other agents for cancer treatment.

#### Combined with PORCN inhibitors

Dual inhibition using PORCN and PI3K inhibitors effectively suppresses the growth of TNBC and PDAC xenografts by impeding cell proliferation and glucose metabolism.^742^ WNT974, when paired with the tyrosine kinase inhibitor nilotinib (NIL), significantly augments the inhibition of proliferation and colony-forming capacity of CML stem and progenitor cells and further diminishes the growth of these cells in immunodeficient mice compared to NIL alone.^675^ In preclinical models of EOC, WNT974 combined with paclitaxel demonstrates enhanced anti-tumor activity.^676^ Additionally, the combination of another PORCN inhibitor, ETC-159, with the PI3K inhibitor GDC-0941, has been shown to reduce the proliferation and growth of RNF43 mutant pancreatic cancer xenografts in vivo.^743^

#### Combined with β-catenin/TCF inhibitors

Mologni and colleagues found that the β-catenin/TCF inhibitors PKF115-584 and pyrvinium pamoate effectively blocked β-catenin-dependent transcription and synergized with a KRAS inhibitor in colon cancer cells driven by Wnt and KRAS oncogenic signaling.^744^ However, this combination was ineffective in colon cancer cells harboring BRAF mutations. The combined treatment outperformed monotherapy in inducing cell cycle arrest, apoptosis, downregulation of MYC and survivin, and inhibition of anchorage-independent growth.^745,746^

#### Combined with Tankyrase inhibitors

Furthermore, studies on CRC using Tankyrase inhibitor NVP-TNKS656, combined with AKT and PI3K inhibitors, in both mouse xenografts and patient-derived spheroids, observed a reduction in nuclear β-catenin levels, which correlated with increased apoptosis, suggesting that Tankyrase inhibitors may overcome resistance to AKT and PI3K inhibitors.^691^ In head and neck squamous cell carcinoma, combination treatment with cisplatin and Tankyrase inhibitor XAV-939 enhances cytotoxicity, eradicates cancer stem-like cell phenotypes, and improves chemotherapy sensitivity.^747^ In vitro and in vivo studies with IWR-1, another Tankyrase inhibitor, demonstrated that IWR-1 induces apoptosis in osteosarcoma spheroid cells and, when combined with doxorubicin, exhibits synergistic cytotoxicity, effectively reversing doxorubicin resistance. Co-administration of IWR-1 and doxorubicin in vivo significantly reduced tumor progression, associated with specific downregulation of TCF/LEF transcriptional activity, nuclear β-catenin, and the CSC marker Sox2.^701^

#### Combined with other small molecule inhibitors

Other small molecule inhibitors have shown potential in combination therapies; for instance, dual inhibition of CK2/TNIK kinase may overcome paclitaxel resistance in TNBC. ICG-001 alone substantially inhibited both anchorage-dependent and -independent growth of various PDAC lines, and its combination with gemcitabine further enhanced growth inhibition in vitro.^710^ Moreover, the combined use of Pyrvinium and paclitaxel significantly curbed tumor growth.^706^ Knockdown of the Wnt pathway transcription factor SOX4 in BT-549 cells resulted in reduced proliferation and migration, while combined treatment with iCRT-3 and SOX4 knockdown synergistically inhibited cell proliferation and induced apoptosis.^727^

#### Combined with monoclonal antibodies

Beyond the promising results with small molecule inhibitors, combination therapies involving monoclonal antibodies also hold significant clinical potential.^719^ Phase 1 clinical trials of OMP-18R5 in combination with docetaxel, paclitaxel, and albumin-bound paclitaxel (Abraxane) plus gemcitabine are underway in patients with NSCLC, BC, and pancreatic cancer, respectively. Similarly, Phase 1b trials of OMP-54F28 in combination with sorafenib, paclitaxel plus carboplatin, and nab-paclitaxel plus gemcitabine are ongoing in patients with liver cancer, OC, and pancreatic cancer, respectively.^746,748,749^ Notably, pretreatment with OMP-54F28 has shown synergistic effects with taxanes.^745,746^

### Challenges of targeted therapies on Wnt/β‑catenin signaling

The Wnt signaling pathway’s involvement in oncogenesis and various diseases has emerged as a pivotal area of research, presenting an intriguing avenue for therapeutic intervention. Nevertheless, the development of therapies targeting this pathway remains in its early stages. The Wnt pathway’s essential role in the normal functioning of adult cells poses significant challenges in the development of targeted therapies, particularly concerning toxicity and off-target effects. Despite the initiation of clinical trials for a range of hematological and solid malignancies, no drugs targeting the Wnt pathway have yet received approval. This pathway is essential for the maintenance of stem cells and the regeneration of tissues and organs, and its inhibition can negatively impact Wnt-dependent stem cell populations, such as those involved in skin metabolism. Early studies on tankyrase inhibitors have highlighted significant gastrointestinal toxicity at high doses, which constrains their clinical applicability. Additionally, the successful clinical deployment of CRT inhibitors faces substantial hurdles, largely due to the challenge of identifying compounds that can selectively modulate the nuclear transcriptional activity of β-catenin without disrupting its pivotal role in stabilizing adherens junctions at the cell membrane. Moreover, given the Wnt pathway’s regulation of various aspects of bone formation, agonists have been investigated to enhance bone growth; however, an unintended consequence of Wnt inhibition is the elevation of bone turnover markers. Despite these obstacles, the potential of the Wnt signaling pathway as a therapeutic target remains promising. Ongoing, rigorous research into the pathway’s mechanisms and its dual roles in both normal physiological and pathological processes is anticipated to yield targeted treatments with an improved safety profile. Achieving this objective holds the promise of delivering transformative breakthroughs in medical science.

## Conclusion and perspectives

The Wnt signaling pathway is a highly conserved mechanism fundamental to cell proliferation, differentiation, and migration. It consists of key components such as Wnt ligands, Fzd receptors, co-receptors LRP5/6, downstream regulatory proteins (including Dsh/Dvl and Axin), β-catenin, and TCF/LEF transcription factors. Furthermore, upstream signaling molecules such as FOXP4, NR2E3, YTHDF2, and various lncRNAs within the Wnt signaling cascade play pivotal roles in influencing the onset and progression of numerous diseases. Upon activation, Wnt ligands engage with the Fzd receptor and LRP5/6, leading to the activation of Dsh/Dvl, which in turn inhibits GSK3β. This inhibition results in the stabilization and accumulation of β-catenin in the cytoplasm, allowing it to translocate to the nucleus. Once in the nucleus, β-catenin associates with TCF/LEF transcription factors to modulate the expression of target genes. A thorough understanding of the molecular mechanisms underpinning the Wnt signaling pathway, including receptor-ligand interactions, downstream signal transduction, and negative feedback loops (such as those involving Axin2 and DKK), is essential for elucidating its functional dynamics across different cellular contexts. Additionally, investigating the distinct roles of non-canonical Wnt signaling pathways, such as Wnt/PCP and Wnt/Ca^2+^, and their crosstalk with canonical Wnt signaling, presents vast opportunities for future research. Recent studies have identified several novel regulators of Wnt/β-catenin signaling.^132^ Twa1/Gid8 functions as a nuclear retention factor for β-catenin within the context of Wnt signaling and colorectal tumorigenesis.^750^ In the absence of Wnt signaling, Twa1 is integrated into the axin complex with β-catenin, leading to its ubiquitination and degradation. Upon activation of Wnt signaling, Twa1 translocates to the nucleus, where it binds to and retains β-catenin.^751^ FOXK1 and FOXK2, authentic Dvl-interacting proteins, enhance Wnt/β-catenin signaling by facilitating the nuclear import of Dvl. USP7, a potent negative regulator of Wnt/β-catenin signaling, interacts directly with Axin *via* its TRAF domain, promoting Axin deubiquitination and stabilization. Inhibition of USP7 augments Wnt/β-catenin signaling, thereby influencing the differentiation of osteoblasts and adipocytes.^752^ Additional key regulators of this pathway include ICAT, Kdm2a/b, Dapper1, and GPR177.^753–756^

This review delves into the intricate interactions between the Wnt signaling pathway and other key signaling pathways, highlighting their complexity and diversity. The Wnt and TGF-β/BMP pathways orchestrate gene expression through the interplay of Smad and β-catenin, while the Wnt and Notch pathways collaboratively regulate cell differentiation. For example, in intestinal stem cells, Notch signaling suppresses Wnt activity, driving differentiation into absorptive cells. The Wnt and PI3K/Akt pathways, through Akt activation, promote cell survival and proliferation, jointly managing cellular metabolic processes. Interactions between key elements of the Wnt/β-catenin signaling pathway and NF-κB profoundly affect inflammatory processes and immune responses. A profound understanding of these interactions offers valuable insights into both normal and pathological cellular functions. Aberrant Wnt signaling is a hallmark of diseases, including cancer, where increased β-catenin stability triggers the activation of oncogenes such as c-Myc and Cyclin D1, thereby promoting cancer cell proliferation and tumor growth. Additionally, Wnt signaling influences tumor invasion and metastasis by modulating the behavior of stromal cells within the cancer microenvironment. It also contributes to immune evasion, facilitating tumor cell survival and dissemination by altering the tumor immune landscape. A wide range of degenerative genetic disorders are linked to mutations in components of the Wnt signaling pathway, originating from either somatic cell alterations or hereditary transmission. The non-canonical Wnt signaling pathway, which functions *via* receptor-mediated mechanisms and the activation of second messengers such as RAC1, JNK, Ca^2+^-dependent CaMKII, and PKC, has been causally implicated in the development of vascular and myocardial diseases, as demonstrated by both animal and human experimental models. Potential therapeutic strategies targeting the Wnt pathway include small molecule inhibitors, monoclonal antibodies, and combination therapies. These approaches aim to directly inhibit β-catenin accumulation and its transcriptional activity, block signaling through Wnt ligands and receptor antagonists (such as DKK1 and sFRP), combine with immune checkpoint inhibitors to enhance antitumor immune responses, and integrate with other pathway inhibitors to optimize therapeutic outcomes. However, the complexity and redundancy of the Wnt signaling pathways pose significant challenges for research and targeted therapies. The role of Wnt signaling varies across different tissues and cell types, necessitating tailored therapeutic strategies for specific disease types and microenvironments. Given the critical function of Wnt signaling in maintaining normal cell processes and tissue homeostasis, direct inhibition risks severe toxicity and side effects, including reduced bone density and intestinal dysfunction. The development of Wnt pathway inhibitors with high specificity and minimal toxicity remains particularly challenging, especially when targeting protein-protein interactions and intracellular signaling mechanisms. Moreover, tumor cells may develop resistance to Wnt inhibitors by upregulating alternative pathways or acquiring mutations, underscoring the need for innovative combination therapies and strategies to overcome resistance. The Wnt signaling pathway is pivotal in cell biology and disease pathogenesis, and its complex composition and extensive crosstalk with other pathways continue to be a central focus of research. Despite the recognized importance of Wnt signaling in disease progression, therapeutic strategies face substantial challenges, necessitating further in-depth research. This comprehensive review aims to enhance the understanding of the Wnt pathway among researchers, fostering the development of more effective and less toxic treatments, ultimately leading to improved outcomes for patients.
